# African Swine Fever Virus EP364R and C129R Target Cyclic GMP-AMP To Inhibit the cGAS-STING Signaling Pathway

**DOI:** 10.1128/jvi.01022-22

**Published:** 2022-07-21

**Authors:** Niranjan Dodantenna, Lakmal Ranathunga, W. A. Gayan Chathuranga, Asela Weerawardhana, Ji-Won Cha, Ashan Subasinghe, Nuwan Gamage, D. K. Haluwana, YongKwan Kim, WeonHwa Jheong, Haryoung Poo, Jong-Soo Lee

**Affiliations:** a College of Veterinary Medicine, Chungnam National University, Daejeon, Republic of Korea; b Wildlife Disease Response Team, National Institute of Wildlife Disease Control and Prevention, Incheon, Republic of Korea; c Infectious Disease Research Center, Korea Research Institute of Bioscience and Biotechnology, Daejeon, Republic of Korea; Lerner Research Institute, Cleveland Clinic

**Keywords:** African swine fever virus, C129R, EP364R, phosphodiesterase, 2′,3′-cGAMP

## Abstract

African swine fever virus (ASFV) is a highly pathogenic swine DNA virus with high mortality that causes African swine fever (ASF) in domestic pigs and wild boars. For efficient viral infection, ASFV has developed complex strategies to evade key components of antiviral innate immune responses. However, the immune escape mechanism of ASFV remains unclear. Upon ASFV infection, cyclic GMP-AMP (2′,3′-cGAMP) synthase (cGAS), a cytosolic DNA sensor, recognizes ASFV DNA and synthesizes the second messenger 2′,3′-cGAMP, which triggers interferon (IFN) production to interfere with viral replication. In this study, we demonstrated a novel immune evasion mechanism of ASFV EP364R and C129R, which blocks cellular cyclic 2′,3′-cGAMP-mediated antiviral responses. ASFV EP364R and C129R with nuclease homology inhibit IFN-mediated responses by specifically interacting with 2′,3′-cGAMP and exerting their phosphodiesterase (PDE) activity to cleave 2′,3′-cGAMP. Particularly notable is that ASFV EP364R had a region of homology with the stimulator of interferon genes (STING) protein containing a 2′,3′-cGAMP-binding motif and point mutations in the Y76S and N78A amino acids of EP364R that impaired interaction with 2′,3′-cGAMP and restored subsequent antiviral responses. These results highlight a critical role for ASFV EP364R and C129R in the inhibition of IFN responses and could be used to develop ASFV live attenuated vaccines.

**IMPORTANCE** African swine fever (ASF) is a highly contagious hemorrhagic disease in domestic pigs and wild boars caused by African swine fever virus (ASFV). ASF is a deadly epidemic disease in the global pig industry, but no drugs or vaccines are available. Understanding the pathogenesis of ASFV is essential to developing an effective live attenuated ASFV vaccine, and investigating the immune evasion mechanisms of ASFV is crucial to improve the understanding of its pathogenesis. In this study, for the first time, we identified the EP364R and C129R, uncharacterized proteins that inhibit type I interferon signaling. ASFV EP364R and C129R specifically interacted with 2′,3′-cGAMP, the mammalian second messenger, and exerted phosphodiesterase activity to cleave 2′,3′-cGAMP. In this study, we discovered a novel mechanism by which ASFV inhibits IFN-mediated antiviral responses, and our findings can guide the understanding of ASFV pathogenesis and the development of live attenuated ASFV vaccines.

## INTRODUCTION

African swine fever virus (ASFV) is a large double-stranded, cytoplasmic DNA arbovirus belonging to the genus *Asfivirus* in the family *Asfarviridae* (1, 2). The genomic size of ASFV is approximately 170 to 193 kbp, and the genome encodes 150 to 167 proteins that play roles in virus structure formation, viral replication, and immune evasion. However, many viral proteins have unknown functions (3–5). ASFV replicates mainly in the cytoplasm of monocyte- and macrophage-lineage cells (6), where replication predominates in the perinuclear cytoplasmic region, called the viral factory (7). African swine fever has caused headlines with a surge in cases worldwide. This highly contagious hemorrhagic viral disease in pigs has a mortality rate of nearly 100% and threatens the global pork supply and food security (8, 9). However, no effective drugs or vaccines are commercially available for this deadly disease (7).

When a DNA virus infects a permissive cell, viral DNA is released into the cell cytoplasm before viral protein synthesis. Although various cytosolic DNA sensors have been identified, mainly cytosolic viral DNA is recognized by cyclic GMP-AMP (cGAMP) synthase (cGAS), which allows the rearrangement of the cGAS catalytic pocket for the subsequent binding of ATP and GTP as cGAS substrates for the synthesis of 2′,3′ cyclic GMP-AMP (2′,3′-cGAMP) (10, 11). The synthesis of 2′,3′-cGAMP is a crucial first step in initiating cGAS-mediated downstream signaling (12, 13). Synthesized 2′,3′-cGAMP acts as a second messenger that can bind to the endoplasmic reticulum (ER) membrane adaptor-stimulator of interferon gene (STING) (also called MITA, ERIS, and MPYS) and induces conformational changes, activating STING (12). Activated STING then migrates from the ER to the ER-Golgi intermediate compartment (ERGIC), and upon reaching ERGIC and Golgi compartments, STING recruits TANK-binding kinase 1 (TBK1), which phosphorylates the interferon regulatory factor 3 (IRF3). Phosphorylated IRF3 dimerizes and enters the nucleus, leading to the induction of type I interferons (IFNs) and other antiviral genes (14, 15). In contrast, STING activates the inhibitor of nuclear factor-κB (IκB) kinase to release NF-κB, which translocates to the cell nucleus and activates the transcription of proinflammatory cytokine-related genes (16, 17).

The type I IFN response is the first-line defense mechanism against invading viruses, including ASFV (18). Therefore, viruses have evolved diverse antagonistic strategies to evade the type I IFN response and facilitate rapid replication in host cells (19). The virulent ASFV strain Armenia/07 has been shown to inhibit IFN-β production via the cGAS-STING pathway (20), and ASFV has been shown to be sensitive to type I and II IFNs (21). Thus, ASFV also employs various immune evasion mechanisms that regulate various steps in the type I IFN signaling pathways. For instance, ASFV MGF505-7R degrades STING through the autophagy lysosomal pathway (22) and inhibits IRF3 nuclear translocation via IRF3 interaction (23). ASFV DP96R also interferes with the cGAS-STING-TBK1 axis (24). E120R was shown to interact with IRF3 to inhibit the interaction between TBK1 and IRF3 (25). In contrast, ASFV I215L (E2 ubiquitin-conjugating enzyme) impairs IFN-β via the K63-linked ubiquitination of TBK1 and NF-κB signaling (26, 27). Consequently, various ASFV proteins are involved in immune-suppressive mechanisms along with diverse target molecules in type I IFN and NF-κB signaling (28–32). However, the exact mechanism by which the ASFV protein targets cGAMP in type I IFN signaling is unknown.

In this study, we show for the first time that ASFV C129R and EP364R act as specific negative regulators of cGAS-STING signaling by targeting 2′,3′-cGAMP through its nuclease activity. Our findings reveal a novel immune evasion mechanism of ASFV and suggest that the C129R and EP364R genes can be used as new candidate genes for the development of live attenuated ASFV vaccines.

## RESULTS

### EP364R and C129R inhibit 2′,3′-cGAMP-induced IFN-β promoter activity.

Viruses have evolved various strategies to inhibit type I IFN signaling, allowing them to replicate efficiently in the host. To investigate the potential role of ASFV proteins in modulating cGAS-STING-mediated type I IFN signaling, we individually tested 158 ASFV viral proteins by using a luciferase reporter gene assay system. Human embryonic kidney (HEK293T) cells were transfected with IFN-β, TK-Renilla, and Flag-tagged STING plasmids, and each ASFV plasmid was tagged with hemagglutinin (HA). To identify ASFV genes that target genes upstream of STING, we selected 60 genes with little or no reduction in IFN-β promoter activity induced by STING. Next, we performed a 2′,3′-cGAMP-induced IFN-β luciferase assay using 293-Dual hSTING-A162 cells. EP364R and C129R significantly reduced 2′,3′-cGAMP-mediated IFN-β promoter activity (see Fig. S1A in the supplemental material). Thus, we confirmed that ASFV EP364R and C129R inhibited the cGAS-mediated IFN-β promoter activity and potentially reduced 2′,3′-cGAMP at the cellular level.

### EP364R and C129R negatively regulate antiviral immune responses.

**FIG 1:**
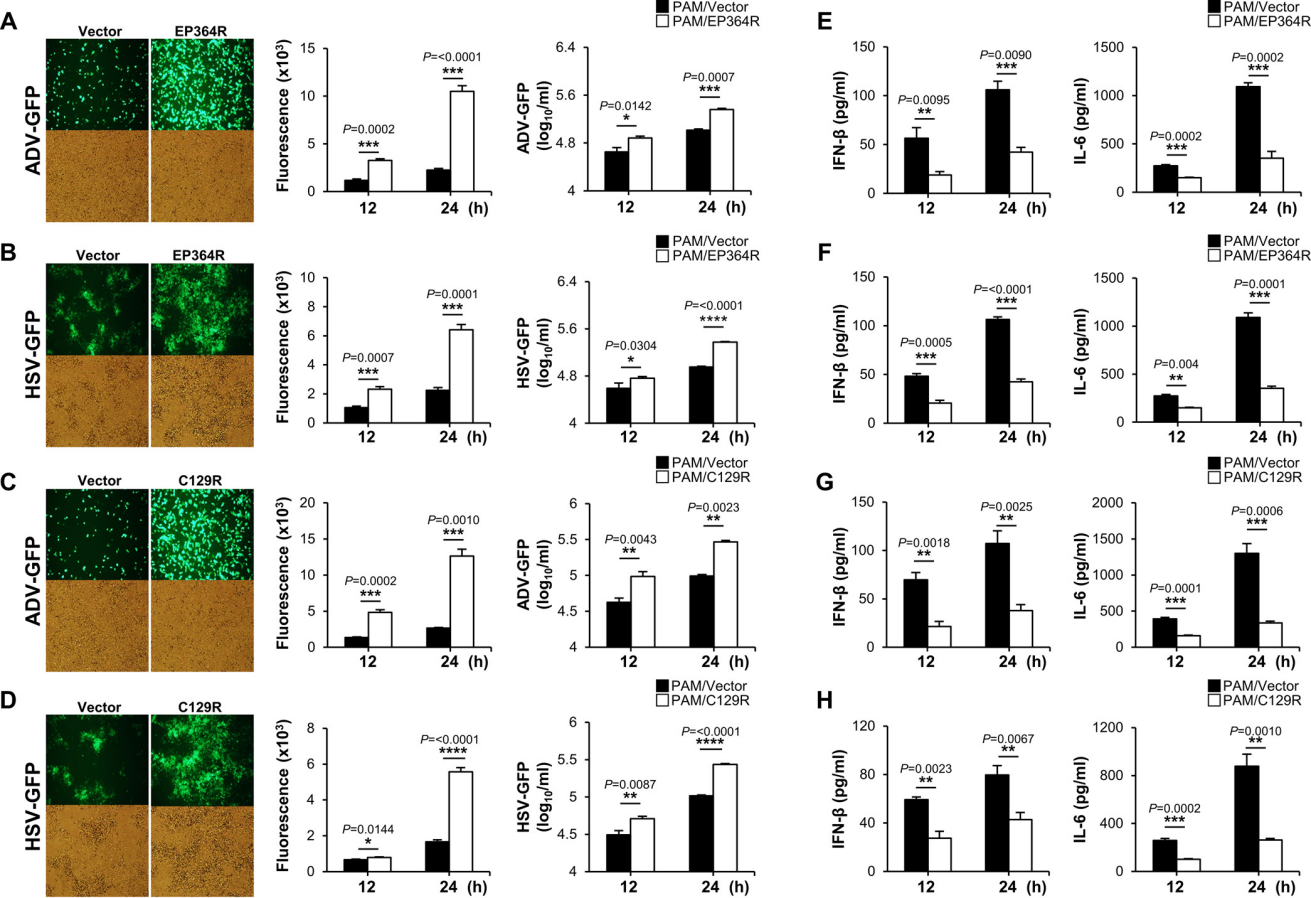
EP364R and C129R negatively regulate antiviral immune responses. PAMs were transiently transfected with Flag-EP364R (A and B) or Flag-C129R (C and D) plasmids with Flag control vector followed by ADV-GFP (MOI = 1) (A and C) infection and HSV-GFP (MOI = 1) (B and D) infection. Viral replication was determined at 24 hpi by GFP expression levels using fluorescence microscopy and quantified at 12 and 24 hpi by a fluorescence modulator. Virus titers of each sample were determined by plaque assay in A549 and Vero cells. Porcine IFN-β and IL-6 secretions in the cell culture supernatant, at 12 hpi and 24 hpi, were determined by ELISA (E to H). Data represent at least two independent experiments, each with similar results, and the values are expressed as means and SD for three biological replicates. Student's *t* test: *, *P* < 0.05; **, *P* < 0.01; ***, *P* < 0.001; ****, *P* < 0.0001.

To further investigate the critical role of ASFV EP364R and C129R on antiviral immune response and virus replication, we transiently transfected Flag-tagged EP364R or C129R into porcine alveolar macrophages (PAMs), porcine kidney epithelial cells (PK-15), and monkey kidney epithelial (MA-104) cells. Each cell was infected with two surrogate viruses, green fluorescent protein-expressing adenovirus (ADV-GFP) or herpes simplex virus (HSV-GFP), in order to replace ASFV. Both viruses are sensitive to interferons, as is ASFV (33, 34). The efficiency of the transient transfection of plasmids in each cell line was confirmed by immunoblotting or quantitative reverse transcription PCR (qRT-PCR) (Fig. S2A to D). Notably, we found that virus replication in EP364R- or C129R-overexpressing cells was significantly higher than that in control cells in the three cell lines (Fig. 1A to D; Fig. S3A to D and S4A to D). Next, we measured the amounts of IFN-β and interleukin 6 (IL-6) secreted from each virus-infected or poly(dA-dT)-transfected cell by using enzyme-linked immunosorbent assay (ELISA). Consistent with the results of virus replication, our results demonstrated that EP364R- or C129R-overexpressing cells secreted fewer cytokines than control cells did (Fig. 1E to H; Fig. S2E and F, S3E to H, and S4E to H). These results suggest that EP364R and C129R negatively regulate the production of type I IFNs and proinflammatory cytokines and enhance DNA virus replication in porcine macrophages and epithelial cells.

### EP364R and C129R inhibit the transcription of antiviral genes and cGAS-STING pathway signaling.

**FIG 2:**
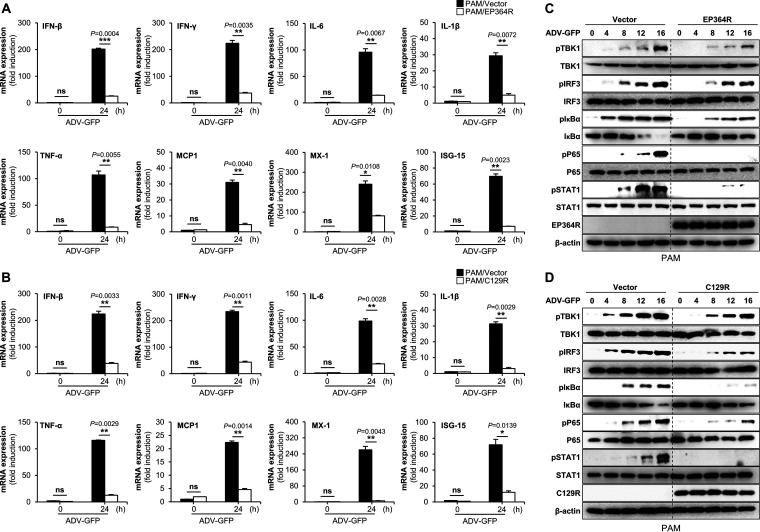
EP364R and C129R inhibit the transcription of antiviral genes and cGAS-STING pathway signaling. (A and B) PAMs were transfected with Flag-EP364R or Flag-C129R plasmids along with Flag control plasmid. Cells were infected with ADV-GFP (MOI = 1) at 24 hpt; cells were harvested at the indicated time points, and total RNA was extracted. Transcription of the indicated genes mRNA was analyzed by qRT-PCR. (C and D) Flag-EP364R or Flag-C129R and Flag control plasmid-transfected PAMs were infected with ADV-GFP (MOI = 1) and cells harvested following the infection at indicated time points. EP364R or C129R gene expression and total and phosphorylated TBK1, IRF3, IκBα, p65, and STAT1 were measured by immunoblotting. β-Actin was used as a loading control indicator. All qPCR data are representative of at least two independent experiments, each with similar results, and the values are expressed as the means and SD for two biological replicates. All the immunoblot data are representative of at least two independent experiments, each with similar results. Student's *t* test: *, *P* < 0.05; **, *P* < 0.01; ***, *P* < 0.001; ns, not significant.

To further evaluate the IFN-antagonistic role of ASFV EP364R and C129R, we analyzed the effects of both proteins on the transcription of interferon genes (IFN-β and IFN-γ), proinflammatory cytokine genes (IL-6, IL-1β, and tumor necrosis factor alpha [TNF-α]) and IFN-stimulated genes (MCP-1, MX-1, and ISG-15). For this experiment, we infected EP364R- or C129R-overexpressing PAMs and PK-15 cells with ADV-GFP and performed qPCR using specific primers (Table S1). The expression of mRNA encoding IFN-β and IFN-γ and other antiviral genes was significantly lower in EP364R- and C129R-overexpressing PAMs (Fig. 2A and B) and PK-15 cells (Fig. S5A and B) than in the control cells. To determine the effect of EP364R and C129R on the antiviral signaling cascade, we investigated the DNA virus-mediated phosphorylation of TBK1, IRF3, IκBα, P65, and STAT1 in PAM and PK-15 cells. EP364R- and C129R-overexpressing PAMs and PK-15 cells were infected with ADV-GFP, and phosphorylation of virus-related signaling molecules and NF-κB-related signaling molecules was assessed at the indicated time points after infection. The phosphorylation levels of TBK1, IRF3, IκBα, P65, and STAT1 were significantly lower in EP364R- and C129R-overexpressing PAMs (Fig. 2C and D) and PK-15 cells (Fig. S5C and D) than in the control cells. These results suggest that EP364R and C129R negatively regulate the host type I IFN signaling pathway and suppress antiviral gene transcription induced by viral infection.

### EP364R and C129R target 2′,3′-cGAMP.

**FIG 3:**
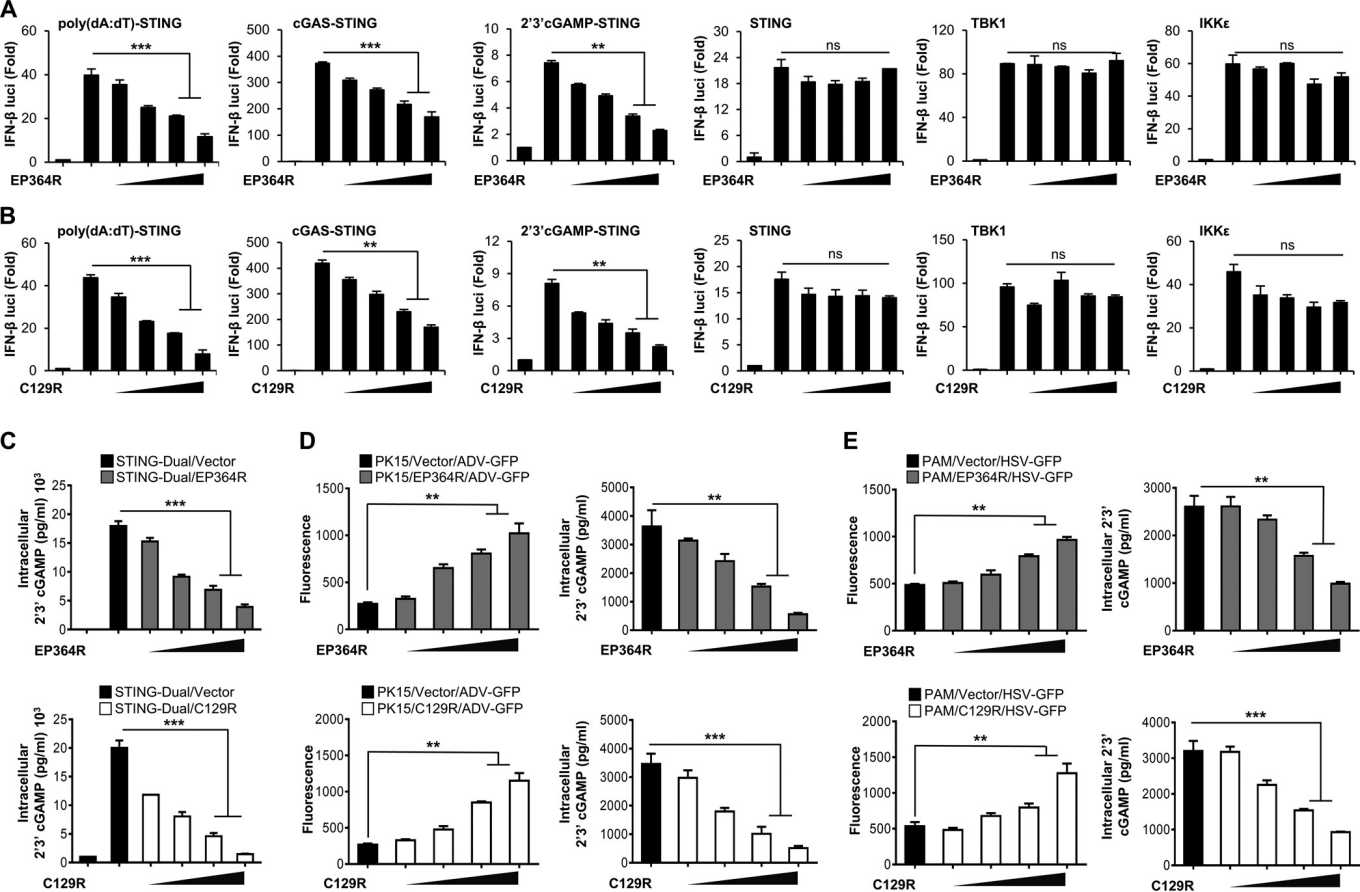
EP364R and C129R target intracellular 2′,3′-cGAMP for degradation. (A and B) HEK293T cells were transfected with Flag-EP364R or Flag-C129R plasmids as indicated along with a firefly luciferase reporter plasmid encoding the IFN-β promoter, the TK-Renilla plasmid as a transfection control to normalize firefly luciferase activity, and expression plasmids of STING, TBK1, and IKKε for 24 h. STING-overexpressing 293-Dual hSTING-A162 cells were used for poly(dA-dT)-, cGAS-, and cGAMP-induced luciferase assays. 293-Dual hSTING-A162 cells were transfected by both Flag-EP364R and Flag-C129R plasmids as indicated along with 3×Flag-cGAS expression plasmids or transfected 2′,3′-cGAMP or poly(dA-dT) for 12 h. After 36 h of transfection, luciferase activity of each sample was measured. Results are expressed relative to those with *Renilla* luciferase alone (internal control). Data represent at least two independent experiments, each with similar results. (C) 293-Dual hSTING-A162 cells were cotransfected with Flag-EP364R or Flag-C129R dose dependently with 3×Flag-cGAS plasmid with vector as a transfection control. (D and E) PK-15 cells and PAMs were transfected with Flag-EP364R and Flag-C129R at increasing doses with Flag control vector as a transfection control and then infected with ADV-GFP (MOI = 1) (D) and HSV-GFP (E) at 24 hpt. 293-Dual hSTING-A162 cells were harvested 24 hpi, and PK-15 cells and PAMs were harvested 4 hpi; cells were analyzed for intracellular 2′,3′-cGAMP by 2′,3′-cGAMP ELISA. Data in panels A and B are representative of three independent experiments, each with similar results, and all the values are expressed as means and SD for two biological replicates. All the ELISA and GFP absorbance data are representative of at least two independent experiments, each with similar results, and the values are expressed as means and SD for three biological replicates. Student's *t* test: **, *P* < 0.01; ***, *P* < 0.001; ns, not significant.

To identify the specific target of the cGAS-STING signaling cascades regulated by ASFV EP364R and C129R, we performed a luciferase promoter assay by coexpressing both genes with several IFN-related genes. We found that both ASFV genes reduced poly(dA-dT)-, cGAS-, and 2′,3′-cGAMP-mediated activation of the IFN-β promoter and NF-κB activities in a dose-dependent manner (Fig. 3A and B; Fig. S6A and B). However, there were no detectable changes in STING-, TBK1-, and IKKε-mediated promoter activity with the increased expression of ASFV EP364R or C129R. These results suggested that ASFV EP364R and C129R target molecules immediately upstream of STING (e.g., 2′,3′-cGAMP). In this study, we found that ASFV EP364R is a homolog to DNA repair endonuclease XPF (ERCC4) (Fig. S1B), and is related to the principle of Holliday junction resolvase, Mus81, in eukaryotes (6). We also found similarities between ASFV C129R and the exonuclease of Lysinibacillus xylanilyticus by using sequence alignment analysis (Fig. S1C).

Because several viruses, bacteria, and host nucleases for selective 2′,3′-cGAMP cleavage have been identified (35–37), we investigated the possibility that ASFV EP364R and C129R target the second messenger 2′,3′-cGAMP for cleavage. To confirm the possibility of ASFV EP364R- and C129R-mediated cleavage of 2′,3′-cGAMP, we examined the intracellular 2′,3′-cGAMP levels by coexpressing ASFV EP364R or C129R with cGAS plasmids in 293-Dual hSTING-A162 cells. Twenty-four hours after transfection, intracellular 2′,3′-cGAMP levels were measured using 2′,3′-cGAMP ELISA. As shown in Fig. 3C, both ASFV EP364R and C129R strongly reduced cGAS-mediated intracellular 2′,3′-cGAMP levels in a dose-dependent manner. In addition, we transfected PK-15 cells and PAMs with increasing amounts of ASFV EP364R or C129R followed by infection with ADV-GFP (multiplicity of infection [MOI] = 1) or HSV-GFP (MOI = 1), respectively. Intracellular 2′,3′-cGAMP levels were measured after 4 h, and fluorescence was measured after 24 h: virus replication was significantly increased in EP364R- and C129R-overexpressing cells, whereas 2′,3′-cGAMP was decreased in cell lysates (Fig. 3D and E). These results strongly suggest that ASFV C129R and EP364R can inhibit type I IFN signaling by targeting 2′,3′-cGAMP.

### EP364R and C129R suppress extracellular 2′,3′-cGAMP-induced responses.

**FIG 4:**
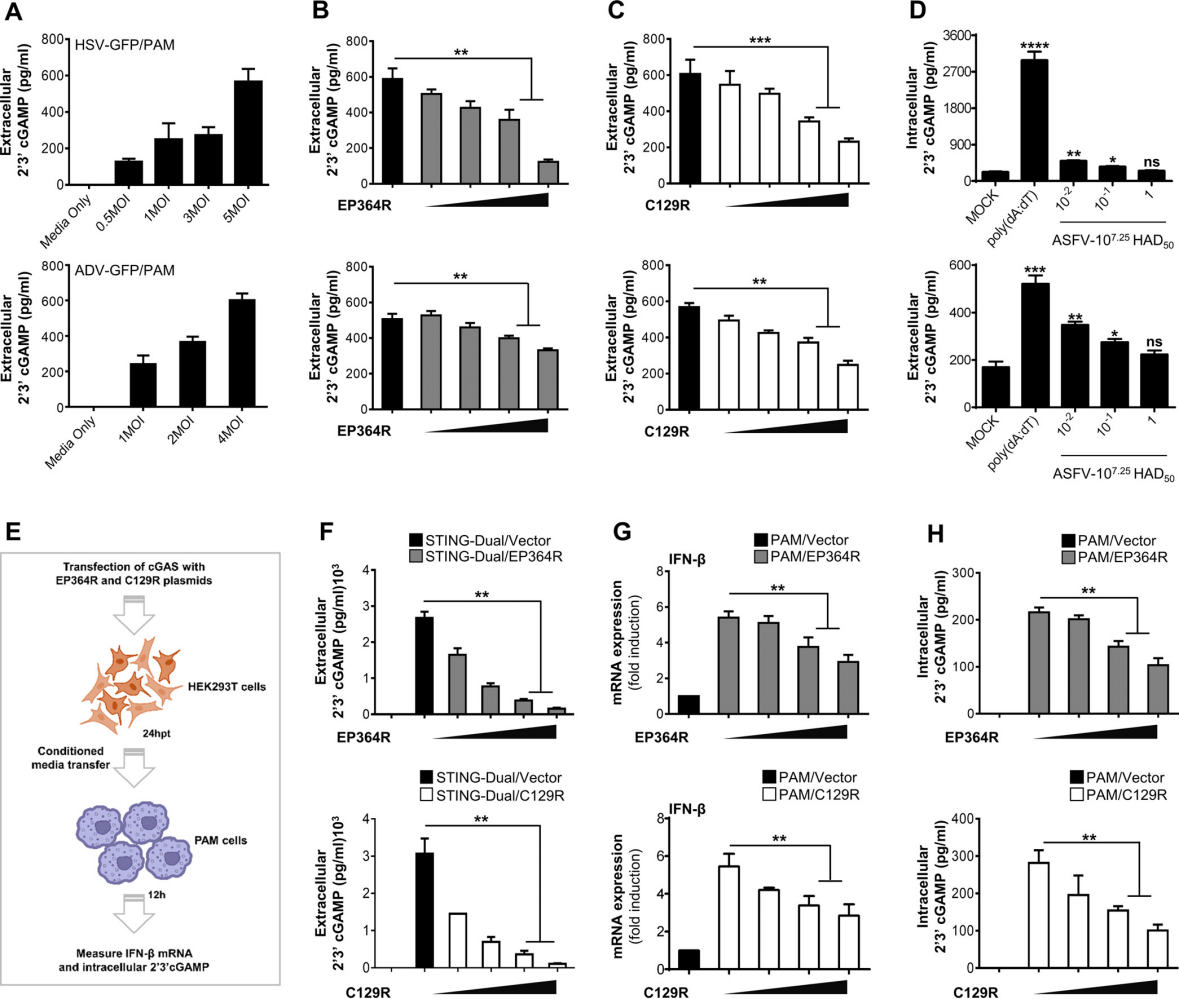
EP364R and C129R degrade 2′,3′-cGAMP and impair its transfer to bystander cells. (A) PAMs were infected with HSV-GFP (MOI = 0.5, 1, 3, and 5) and ADV-GFP (MOI = 1, 2, and 4) for 4 h and 2′,3′-cGAMP level in the supernatant was analyzed using ELISA. (B and C) PAMs were cotransfected with Flag-EP364R (B) or Flag-C129R (C) plasmids dose dependently. Cells were then infected with HSV-GFP (MOI = 5) and ADV-GFP (MOI = 4) for 4 h and assessed the 2′,3′-cGAMP secretion by ELISA. (D) Primary porcine alveolar macrophages were either mock infected or infected with 10-fold-diluted 10^7.25^ HAD_50_ of ASFV-WT as indicated. Poly(dA-dT) was transfected as a positive control. At 5 hpi or 5 hpt, intracellular and extracellular cGAMP levels were measured. (E) Graphical illustration of conditioned-medium transfer experiment. (F) 293-Dual hSTING-A162 cells were cotransfected with Flag-EP364R or Flag-C129R dose dependently with 3×Flag-cGAS plasmid with Flag vector as a transfection control, and the 2′,3′-cGAMP secretion was determined at 24 hpt in the supernatant by ELISA. (G and H) Another 5(F)-independent experiment cell supernatant was collected and immediately transferred to PAMs. After 24 h of incubation, IFN-β transcription (G) and intracellular 2′,3′-cGAMP level (H) were measured compared to untreated control cells. In panel G, data are representative of at least two independent experiments. ELISA and GFP absorbance data are representative of at least two independent experiments, each with similar results, and the values are expressed as means and SD for three biological replicates. Student's *t* test: **, *P* < 0.01; ***, *P* < 0.001; ****, *P* < 0.0001.

Next, we investigated the effects of EP364R and C129R on 2′,3′-cGAMP secretion into the exogenous cellular environment and the activation of IFN gene transcription in bystander PAMs. Zhou et al. (38) found that volume-regulated LRRC8/VRAC channels in cells are critically involved in the host defense against HSV infection by transporting the 2′,3′-cGAMP across the plasma membranes of mouse embryonic fibroblasts (MEFs). Based on this evidence, we hypothesized that the ectopic expression of EP364R or C129R could reduce the transport of 2′,3′-cGAMP into the extracellular spaces of PAMs. First, we infected PAMs with HSV-GFP or ADV-GFP at different MOIs. After 4 h, the released 2′,3′-cGAMP was analyzed using ELISA. The highest MOI of each DNA virus showed an elevation of the 2′,3′-cGAMP concentration in the cell supernatants (Fig. 4A). In contrast, supernatants of cells transfected with EP364R or C129R plasmid showed a dose-dependent reduction of 2′,3′-cGAMP levels (Fig. 4B and C). To investigate whether ASFV infection reduces cGAMP production, primary porcine alveolar macrophages were mock infected or infected with three dilutions (1, 10^−1^, and 10^−2^) of 10^7.25^ 50% hemadsorbing doses [HAD_50_] of ASFV for 5 h. Cells were transfected with poly(dA-dT) for 5 h as a positive control, and intracellularly produced and extracellularly secreted cGAMPs were detected by ELISA. As shown in Fig. 4D, cGAMP production and secretion were dose-dependently decreased after ASFV infection, and poly(dA-dT) enhanced this effect. The decrease in cGAMP levels produced by ASFV may be due to the ASFV EP364R and C129R proteins, or other proteins may be involved in cGAMP degradation. Consequently, the ASFV blocks IFN production by breaking down cGAMP at the cellular level early in infection.

The transmission of secreted 2′,3′-cGAMP to adjacent cells induces type I IFNs (38, 39). To further explain our finding, 293-Dual hSTING-A162 cells were cotransfected with EP364R and cGAS or C129R and cGAS, and 2′,3′-cGAMP secretion after 24 h posttransfection (hpt) was assessed by ELISA (Fig. 4F). Cells transfected with an EP364R or C129R plasmid showed a reduction of extracellular 2′,3′-cGAMP levels in a dose-dependent manner. Cell supernatants of EP364R- or C129R-expressing 293-Dual hSTING-A162 cells were then transferred to PAMs; the transcription of IFN-β (Fig. 4G) and intracellular 2′,3′-cGAMP level (Fig. 4H) were measured 24 h later. Our results showed a gradual dose-dependent inhibition of IFN-β mRNA transcription and intracellular 2′,3′-cGAMP levels in PAMs treated with supernatants of EP364R- or C129R-expressing cells relative to that of cGAS alone. These results suggest that expression of EP364R or C129R can reduce the amount of intracellular 2′,3′-cGAMP and its transport into the extracellular space of cells; consequently, extracellular 2′,3′-cGAMP-induced antiviral responses in bystander cells cannot be activated.

### EP364R and C129R selectively cleave 2′,3′-cGAMP by their phosphodiesterase activity.

**FIG 5:**
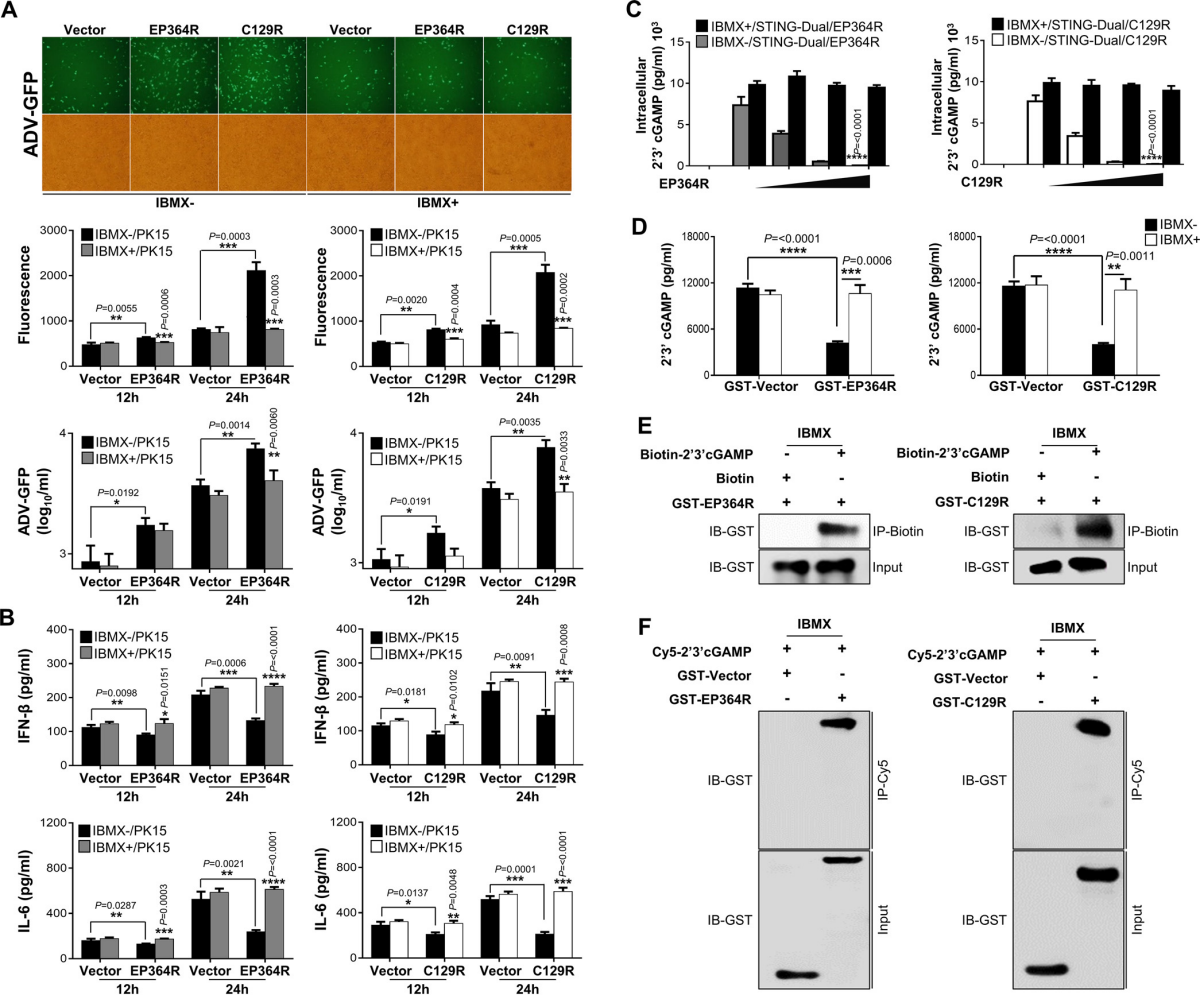
Phosphodiesterase activity of EP364R and C129R inhibits activation of STING by degrading 2′,3′-cGAMP. (A and B) PK-15 cells were transfected with Flag-EP364R and Flag-C129R and treated with 200 nM IBMX at 6 hpt and infected with ADV-GFP (MOI = 1) at 24 hpt. Fluorescence microscopy and fluorescence absorbance and virus replication (A) and IFN-β secretion and IL-6 secretion (B) were measured at indicated time points. (C) 293-Dual hSTING-A162 cells were cotransfected with 3×Flag-cGAS plasmid and indicated plasmids dose dependently, and 6 h before cells were harvested, they were treated with 100 nM IBMX. Cells were harvested 24 hpt, and the intracellular 2′,3′-cGAMP level was measured. (D) *In vitro* 2′,3′-cGAMP degradation assay. Purified GST-EP364R or GST-C129R proteins plus 2.5 μM 2′,3′-cGAMP with or without 1 mM IBMX were incubated for 22 h at 37°C in a reaction mixture. Then 2′,3′-cGAMP level in each sample was quantified by 2′,3′-cGAMP ELISA. (E and F) GST-purified EP364R and C129R protein interaction with biotin-cGAMP or Cy5-cGAMP. (E) Here, 2 μg of ASFV proteins was incubated with 10 μM biotin-cGAMP or 10 μM biotin in a reaction buffer for 2 h at 30°C with the presence of 1 mM IBMX. Biotinylated cGAMP was pulled down by streptavidin magnetic beads followed by immunoblotting with anti-GST antibody. (F) ASFV GST-purified protein with vector was incubated with Cy5-cGAMP and subjected to Cy5 pulldown using anti-Cy5 antibody followed by immunoblotting with anti-GST antibody. All the data are representative of at least two independent experiments, each with similar results, and the values are expressed as means and SD for three biological replicates. All the immunoblot data are representative of at least two independent experiments, each with similar results. Student's *t* test: *, *P* < 0.05; **, *P* < 0.01; ***, *P* < 0.001; ****, *P* < 0.0001.

Our results show that ASFV EP364R and C129R inhibit type I IFN signaling by targeting 2′,3′-cGAMP. Most cyclic dinucleotides (CDNs), including c-di-GMP, c-di-AMP, 3′,3′-cGAMP, and 2′,3′-cGAMP, are cleaved by phosphodiesterases and nucleases (35–37, 40–45). Therefore, we investigated the specific role of nuclease homologs of ASFV EP364R and C129R in the cleavage of 2′,3′-cGAMP containing canonical 3′-5′ and noncanonical 2′-5′ phosphodiester linkages. We first examined ADV-GFP virus replication in the presence of 1-methyl-3-isobutylxanthine (IBMX), a universal phosphodiesterase (PDE) inhibitor. In brief, EP364R- and C129R-expressing PK-15 cells were treated with IBMX (200 nM), followed by ADV-GFP infection. Remarkably, IBMX treatment reduced GFP fluorescence and virus titer (Fig. 5A) while restoring IFN-β and IL-6 levels (Fig. 5B). These data indicate that ASFV EP364R and C129R have phosphodiesterase activities. For further confirmation of the aforementioned PDE activity, increasing amounts of EP364R and C129R were transfected into 293-Dual hSTING-A162 cells with cGAS and treated with 100 nM IBMX for 6 h before the analysis of 2′,3′-cGAMP levels. As expected, there was a robust decrease in the 2′,3′-cGAMP concentration in the cell line with increasing amounts of EP364R or C129R, whereas cells treated with IBMX showed 2′,3′-cGAMP levels similar to those in untreated controls (Fig. 5C). Furthermore, to confirm the cleavage of 2′,3′-cGAMP by ASFV EP364R and C129R, we performed an *in vitro* cleavage assay using Flag-tagged immunoprecipitated proteins and glutathione *S*-transferase (GST)-tagged purified proteins. Before the *in vitro* cleavage assay, expression of Flag-tagged immunoprecipitated proteins was confirmed by immunoblotting (Fig. S6C) and that of GST-tagged purified protein was confirmed by immunoblotting and Coomassie brilliant blue staining (Fig. S7A). GST-tagged EP364R and C129R did not reverse the dose-dependent 2′,3′-cGAMP degradation abilities of EP364R and C129R (Fig. S6D). We performed an *in vitro* cleavage assay using 2′,3′-cGAMP: the 2′,3′-cGAMP levels in the EP364R or C129R samples were significantly lower than those in the control sample, but cleavage was restored in the IBMX-treated samples (Fig. 5D and Fig. S6C).

Importantly, to investigate the critical interaction between ASFV EP364R or C129R and 2′,3′-cGAMP, we prepared Flag-tagged immunoprecipitated or GST-tagged purified EP364R or C129R and synthesized biotin- or Cy5-labeled 2′,3′-cGAMP. To select the optimal IBMX dosage, we first performed a binding assay with Flag-tagged immunoprecipitated EP364R and biotin-labeled 2′,3′-cGAMP by using different doses of IBMX (0.25 mM, 0.5 mM, and 1 mM). We found a progressive binding affinity as the inhibitor concentration increased (Fig. S6E). To further confirm the binding between ASFV EP364R or C129R and 2′,3′-cGAMP, we performed an *in vitro* binding assay using GST-purified EP364R or C129R and 2′,3′-cGAMP labeled with biotin or Cy5 (Fig. 5E and F) and Flag-tagged immunoprecipitated EP364R or C129R and 2′,3′-cGAMP labeled with biotin in the presence of 1 mM IBMX (Fig. S6F). We observed a clear interaction between ASFV EP364R or C129R and 2′,3′-cGAMP in the presence of IBMX. These results suggest that ASFV EP364R or C129R interacts with 2′,3′-cGAMP, and its subsequent cleavage counteracts the cGAS-STING signaling and the generation of type I IFNs.

### EP364R competes with STING to hijack 2′,3′-cGAMP for cleavage.

**FIG 6:**
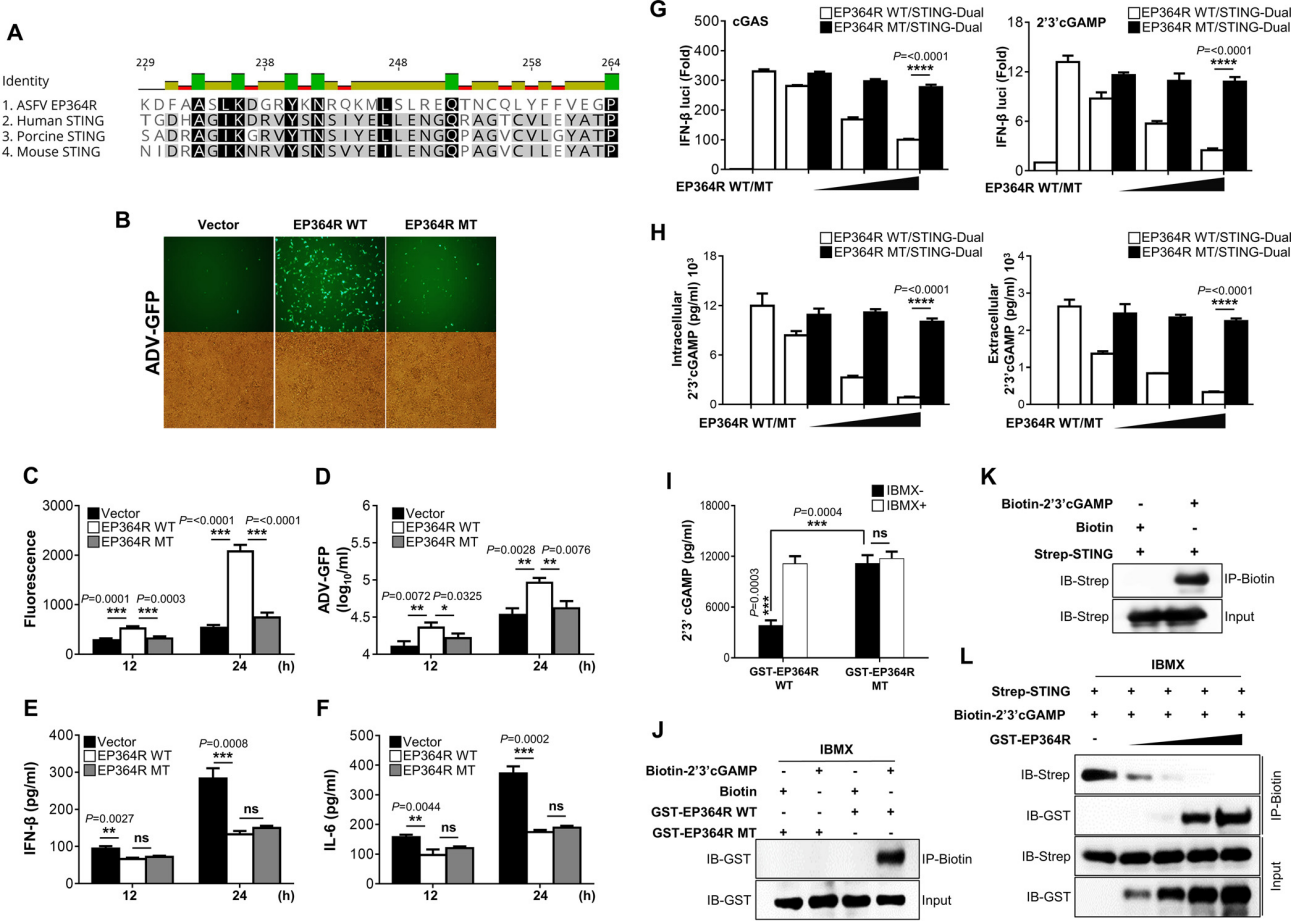
EP364R competes with STING and hijacks 2′,3′-cGAMP. (A) ASFV EP364R and human, porcine, and mouse STING sequence analysis. (B to F) PK-15 cells were transfected with plasmids as indicated and infected with ADV-GFP. Viral replication was determined at 24 hpi by GFP expression levels using fluorescence microscopy (B) and quantified at 12 hpi and 24 hpi by fluorescence modulator (C). (D) A plaque assay in A549 cells determined the virus titers of each sample. (E and F) Porcine IFN-β and IL-6 secretion in cell culture supernatant was determined by ELISA. (G) 293-Dual hSTING-A162 cells were cotransfected with 3×Flag-cGAS and the indicated plasmids. At 24 hpt, IFN-β luciferase activity was measured. Another set of transfected cells were stimulated with 2′,3′-cGAMP ligand at 24 hpt. At 36 hpt, IFN-β luciferase activity was measured. (H) Intracellular and extracellular 2′,3′-cGAMP level was measured from the collected cell lysate and the supernatant from the cGAS luciferase assay. (I) *In vitro* 2′,3′-cGAMP degradation assay. GST-purified proteins EP364R-WT and EP364R-MT were incubated with 2.5 μM 2′,3′-cGAMP with or without 1 mM IBMX for 22 h at 37°C in reaction buffer followed by 2′,3′-cGAMP ELISA. (J) *In vitro* 2′,3′-cGAMP binding assay. GST-purified ASFV EP364R-WT or EP364R-MT proteins (2 μg) were incubated with 10 μM biotin-cGAMP or 10 μM biotin in reaction buffer for 2 h at 30°C with 1 mM IBMX and subjected to biotin pulldown by streptavidin magnetic beads followed by immunoblotting with anti-GST antibody. (K) *In vitro* STING–2′,3′-cGAMP binding assay. Immunoprecipitated Strep-STING protein (2 μg) was incubated with 10 μM biotin–2′,3′-cGAMP or with biotin in reaction buffer for 2 h at 30°C. Samples were subjected to streptavidin pulldown followed by immunoblotting with anti-Strep antibody. (L) *In vitro* 2′,3′-cGAMP binding competition assay. Increasing amounts of GST-purified EP364R-WT protein (0.25 μg, 0.5 μg, 1 μg, and 2 μg) and GST vector protein as a control (1.75 μg, 1.5 μg, and 1 μg) with a constant amount of Strep-STING protein (1 μg) were incubated with 10 μM biotin–2′,3′-cGAMP in reaction buffer for 2 h at 30°C with 1 mM IBMX. The incubated protein mixture was immunoprecipitated by streptavidin followed by immunoblotting with anti-GST and anti-Strep antibodies. All the data are representative of at least two independent experiments, each with similar results, and the values are expressed as the means and SD for three biological replicates. All the immunoblot data are representative of at least two independent experiments, each with similar results. Student's *t* test: *, *P* < 0.05; **, *P* < 0.01; ***, *P* < 0.001; ****, *P* < 0.0001; ns, not significant.

Thus far, our results indicate that ASFV EP364R and C129R each bind to and cleave 2′,3′-cGAMP. Studies have identified specific amino acid residues within STING that interact with 2′,3′-cGAMP (11, 46, 47). Therefore, to identify the specific amino acids of ASFV EP364R and C129R that can interact with 2′,3′-cGAMP, we first compared the amino acid sequences of EP364R and C129R and the 2′,3′-cGAMP-interacting motif of STING by using the alignment program. Surprisingly, we found a similar 2′,3′-cGAMP-interacting motif of STING in the EP364R sequence (Fig. 6A). Based on the literature, we hypothesized that amino acids tyrosine-76 and asparagine-78 of EP364R could interact with 2′,3′-cGAMP for cleavage.

To verify this hypothesis, we constructed Flag- and GST-tagged EP364R mutant (EP364R-MT) plasmids harboring a tyrosine-to-serine mutation at position 76 (Y76S) and an asparagine-to-alanine substitution at position 78 (N78A). First, we balanced the protein expression of wild-type EP364 (EP364R-WT) and EP364R-MT in HEK293T and PK-15 cells (Fig. S7B) and investigated the effect of EP364R-MT on virus replication and IFN production compared with that of EP364R-WT in PK-15 cells. As expected, EP364R-MT lost its ability to inhibit IFN signaling in a manner that decreased viral GFP expression (Fig. 6B and C) and virus replication (Fig. 6D) and increased secretion of IFN-β and IL-6 (Fig. 6E and F). Next, we evaluated cGAS- and 2′,3′-cGAMP-mediated IFN-β luciferase activity using the EP364R-WT and EP364R-MT plasmids. As shown in Fig. 6G, EP364R-MT could not reduce IFN-β luciferase activity. Similarly, EP364R-MT did not reduce intracellular and extracellular 2′,3′-cGAMP levels (Fig. 6H) and did not show 2′,3′-cGAMP cleavage (Fig. 6I and Fig. S7C). We also tested the interaction between GST-tagged EP364R-MT (Fig. 6J) and Flag-tagged (Fig. S7E) protein and biotinylated 2′,3′-cGAMP; EP364R-MT did not interact with 2′,3′-cGAMP.

Based on these results, we hypothesized that ASFV EP364R and STING compete for binding to 2′,3′-cGAMP. To confirm this hypothesis, we performed an *in vitro* competition assay using GST-tagged purified EP364R, streptavidin (Strep)-tagged immunoprecipitated STING and biotin- or Cy5-labeled 2′,3′-cGAMP. First, we confirmed the binding between immunoprecipitated STING and the biotin- or Cy5-labeled 2′,3′-cGAMP (Fig. 6K and Fig. S7D), and we found that the interaction between 2′,3′-cGAMP and STING was reduced in a dose-dependent manner by the EP364R protein (Fig. 6L and Fig. S7F), suggesting that ASFV EP364R inhibits the binding between STING and 2′,3′-cGAMP. These findings indicate that ASFV EP364R has a binding motif similar to STING that interacts with 2′,3′-cGAMP and inhibits STING activation by hijacking 2′,3′-cGAMP.

### EP364R and C129R produce intermediate by-products of 2′,3′-cGAMP cleavage.

**FIG 7:**
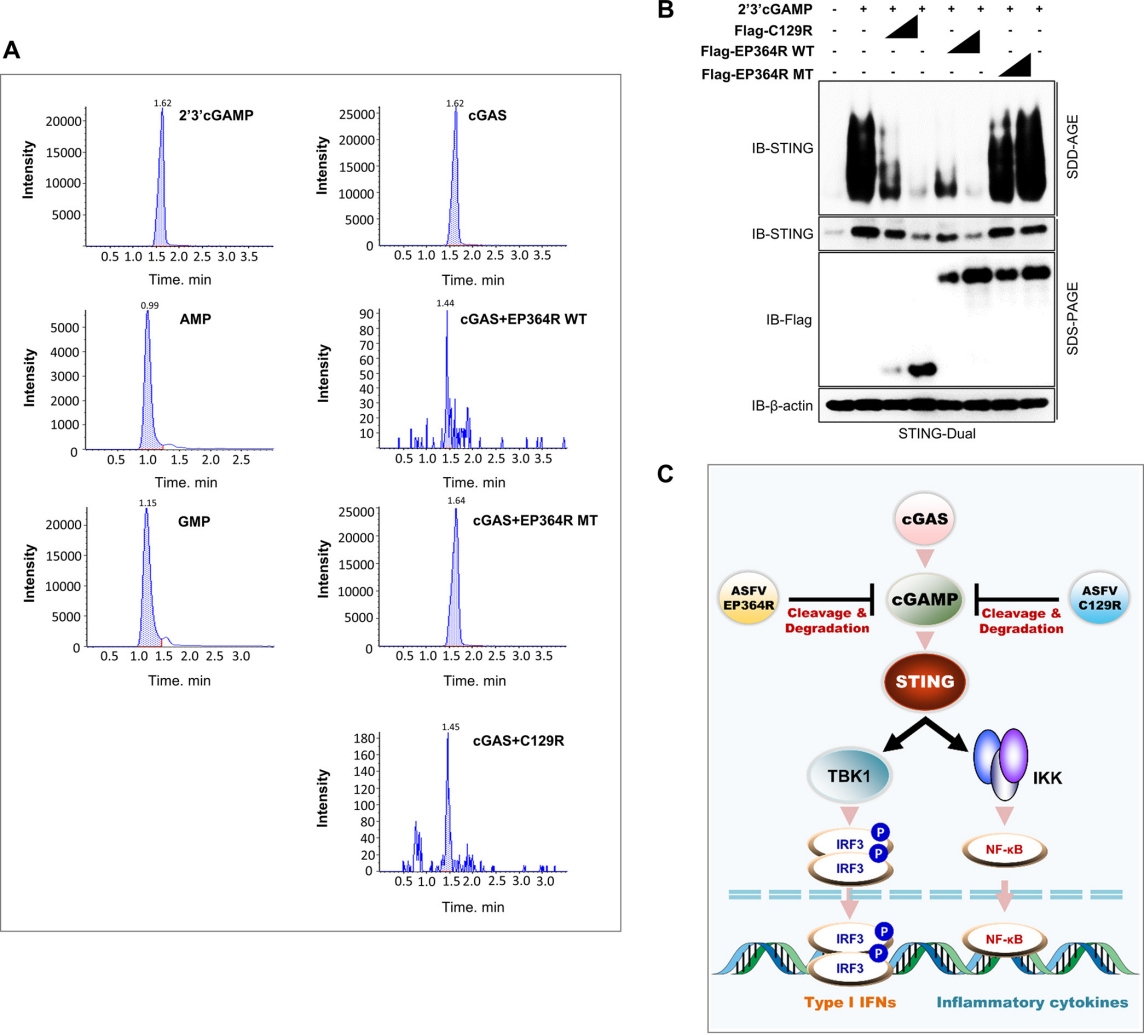
EP364R and C129R produce intermediate by-products of cGAMP degradation. (A) HEK293T cells were cotransfected with Flag-EP364R or Flag-C129R plasmids with 3×Flag-cGAS plasmids for 24 h. Nucleotides of the harvested cell pellet were extracted, and 2′,3′-cGAMP levels were analyzed along with 2′,3′-cGAMP, AMP, and GMP standards using UHPLC. Data are representative of two independent experiments, each with similar results. (B) STING polymerization inhibition SDD-AGE assay. 293-Dual hSTING-A162 cells were cotransfected with plasmids as indicated. At 24 hpt, 8 μg 2′,3′-cGAMP ligand was transfected, and 12 h later, cells were harvested and lysate prepared for the SDD-AGE assay. STING polymerization was detected by immunoblotting with anti-hSTING antibodies. (C) Summary of the immune escape mechanism of ASFV EP364R and C129R proteins.

Finally, to confirm the exact cleavage of 2′,3′-cGAMP, we performed ultra-high-performance liquid chromatography (UHPLC) analysis. HEK293T cells were cotransfected with the cGAS plasmid along with each control, ASFV EP364R-WT, EP364R-MT, and C129R plasmids; next, nucleotides were extracted from the cells. For UHPLC analysis, 2′,3′-cGAMP, GMP, and AMP were used as standards. We found the 2′,3′-cGAMP peaks in cells transfected with cGAS alone and those with the EP364R-MT plasmid, but by-products other than GMP or AMP were observed in cells transfected with the EP364R-WT or C129R plasmid (Fig. 7A). We assumed that the by-products were generated by the marked cleavage of 2′,3′-cGAMP.

### EP364R and C129R inhibit STING activation in 2′,3′-cGAMP treated cells.

Additionally, as previously described, we performed a STING aggregation analysis to investigate the effect of 2′,3′-cGAMP cleavage by ASFV EP364R or C129R on STING activation using the semidenaturing detergent agarose gel electrophoresis (SDD-AGE) assay (48). After transfection of 293-Dual hSTING-A162 cells with two different doses of ASFV EP364R-WT, EP364R-MT, and ASFV C129R plasmids, cells were stimulated with the 2′,3′-cGAMP ligand, and STING aggregation was analyzed by immunoblotting with an anti-hSTING antibody. STING expression and aggregation were inhibited by both genes in a dose-dependent manner, whereas EP364R-MT did not affect the STING activation (Fig. 7B). These results suggest that the cleavage of 2′,3′-cGAMP by ASFV EP364R or C129R inhibits STING activation. In summary, 2′,3′-cGAMP is targeted and degraded by ASFV EP364R or C129R to inhibit STING activation and the cGAS-STING pathway (Fig. 7C).

## DISCUSSION

cGAS, interferon gamma-inducible protein 16 (IFI16), absent in melanoma 2 (AIM2), probable ATP-dependent RNA helicase DDX41, and RNA PolIII-mediated innate immunity are the first lines of defense against DNA viruses (49). The cytosolic DNA sensor cGAS is activated by cytosolic viral DNA and elicits the production of 2′,3′-cGAMP. Synthesized 2′,3′-cGAMP interacts with ER-resident STING and triggers downstream signaling of type I IFNs through TBK1 and IKK, inducing the activation of IRF3 and releasing cytokines, including type I IFNs, the first line of defense against viral infections (50–55).

Cyclic dinucleotides, whose primary role is cellular signal transduction, are indispensable messenger molecules in various organisms (49, 56, 57). CDNs, originally identified as second messengers in bacteria, play a significant role in virulence, motility, metabolism, and survival (58). CDNs containing 3′-5′ internucleotide linkages, namely, canonical c-di-AMP (cAMP-AMP), c-di-GMP (cGMP-GMP), and c-AMP-GMP (3′,3′-cGAMP), are ubiquitous in bacteria, whereas those in mammalian cells (unlike bacterial CDNs with two 3′-5′ bonds) yield 2′,3′-cGAMP, the first reported metazoan messenger molecule produced by cGAS in response to pathogenic DNA (12, 59–62). 2′,3′-cGAMP in mammalian cells is highly stable, and cGAS-triggered horizontal transfer of cGAMP across cellular gap junction channels confers rapid antiviral immunity to neighboring cells (63). Particularly notable is that tumor cells transfer cGAMP through gap junctions and activate STING and type I IFNs, which supports tumor growth and chemoresistance (64, 65). In addition, 2′,3′-cGAMP can be packaged into viral particles and transferred to cells such as macrophages, activating innate immunity and antiviral responses (58, 66). Consequently, 2′,3′-cGAMP transmission between cells is an efficient mechanism for STING activation, now recognized as a critical target of autophagy and inflammasome formation in response to infection by DNA viruses (50, 67, 68).

In this study, we revealed a novel molecular mechanism of ASFV EP364R and C129R that inhibits cellular 2′,3′-cGAMP-mediated antiviral responses. First, we showed that overexpression of both genes increased viral replication, as exhibited by ADV-GFP and HSV-GFP, by inhibiting the virus induced type I IFN signaling cascade and IFN production. Second, based on the results of the sequence homology, the IFN-β luciferase reporter assay and immunoprecipitation assay, ASFV EP364R and C129R target 2′,3′-cGAMP and were confirmed to counteract IFN production. 2′,3′-cGAMP interacts with ASFV C129R and EP364R, and elevated intracellular and extracellular 2′,3′-cGAMP due to viral infection or overexpression of cGAS is degraded by both ASFV genes with phosphodiesterase activity. However, the universal phosphodiesterase inhibitor IBMX inhibited the specific functions of both genes. Third, sequence analysis of EP364R and STING showed that EP364R contains a 2′,3′-cGAMP binding motif, allowing it to compete with STING for 2′,3′-cGAMP and that tyrosine-76 and asparagine-78 of EP364R are required for 2′,3′-cGAMP interaction. Finally, ASFV EP364R and C129R impaired STING activation and self-aggregation upon 2′,3′-cGAMP stimulation. Hence, our findings suggest that ASFV EP364R and C129R downregulate type I IFNs through 2′,3′-cGAMP degradation via their phosphodiesterase activity.

DNA viruses have evolved many strategies to evade host type I IFN responses, predominantly the cGAS-STING axis, and facilitate the successful infection of host cells (19). For the first time, we have shown that the ASFV dose-dependently decreases cGAMP production and secretion at the cellular level in porcine primary alveolar macrophages. STING activation and phosphorylation during ASFV virulent strain Armenia/07 virus infection are completely inhibited than STING phosphorylation during attenuated NH/P68 virus infection, demonstrating that ASFV mainly targets STING and upstream of STING to suppress IFN production (20). Therefore, direct cGAMP clearance or inhibition of cGAMP production by inhibiting cGAS is the main target of ASFV immune evasion. These two ASFV genes may play a major role in the degradation of cGAMP during the ASFV viral infection. Similar to ASFV EP364R and C129R in this study, some viral enzymes that degrade CDNs block the binding of 2′,3′-cGAMP to STING through 2′,3′-cGAMP degradation (69). Eaglesham et al. performed a biochemical screening of 24 mammalian viruses (35) and identified poxvirus nuclease (poxin) as a novel category of a 2′,3′-cGAMP-degrading enzyme that inhibits the STING-dependent type I IFN production (44). Also, studies have confirmed that the 2′-5′ PDE activity of nonstructural 2 protein (NS2) of mouse hepatitis virus (MHV) and VP3 protein of rotavirus (RVA) cleave 2′-5′-oligoadenylate synthetases to increase virus replication in macrophages (70, 71). Additionally, phosphodiesterases of bacterial origin have also been shown to evade the innate immune system by degrading 2′,3′-cGAMP. For example, the Vibrio cholerae EAL domain of PDE (VcEAL) degrades the second messengers c-di-GMP and 2′,3′-cGAMP, and Mycobacterium tuberculosis CdnP degrades 2′,3′-cGAMP (36, 43). Particularly notable is that ectonucleotide pyrophosphatase/phosphodiesterase 1 (ENPP1; also named PC-1), an intracellular enzyme with nucleotide pyrophosphatase and phosphodiesterase enzymatic activities, is a 2′,3′-cGAMP-degrading enzyme with high specificity in mammalian tissues and plasma (72, 73). At the beginning of this study, we found that the ASFV EP364R gene sequence is similar to that of the DNA repair endonuclease XPF (ERCC4)/MUS81 of eukaryotes (6) and that ASFV C129R is homologous to the DNA polymerase/3′-5′ exonuclease PolX of Lysinibacillus xylanilyticus (NCBI reference sequence WP_100542864.1) (Fig. S1B and C). Because nucleases are phosphodiesterases that cleave one of the two connecting phosphodiester bonds at the middle (endonuclease) or 3′ or 5′ end (exonuclease) of a nucleic acid chain (74), and 2′,3′-cGAMP phosphodiesterase is a highly conserved enzyme that catalyzes the hydrolysis of the 3′-5′ CDNs (75), we hypothesized that both ASFV genes exert phosphodiesterase activity to cleave 2′,3′-cGAMP and proved this hypothesis.

Eleven classes of phosphodiesterase (PDE1 to PDE11) cleave the 3′-5′ phosphodiester bond in cAMP and cGMP (76). IBMX is a widely used nonspecific PDE inhibitor that inhibits only phosphodiesterases that cleave the 3′-5′ phosphodiester bond and not the 2′-5′ phosphodiester bonds. IBMX inhibits PDE1, PDE2, PDE3, PDE4, PDE5, PDE7, and PDE11, whereas PDE8A, PDE8B, and PDE9 are insensitive to IBMX (77). In general, IBMX inhibits PDEs and induces cellular cAMP and cGMP levels (78), which activates cyclic-nucleotide-regulated protein kinases (79). In our study, we used IBMX because we found that ASFV EP364R and C129R are homologs of 3′-5′ nucleases that can act as phosphodiesterase enzymes. Although ASFV EP364R and C129R can be PDE8A, PDE8B, or PDE9, Fig. 5 shows that both proteins lose their inhibitory activity after treatment with IBMX, which disrupts phosphodiesterase activity. Therefore, ASFV EP364R and C129R act as phosphodiesterase enzymes that target the 3′-5′ phosphodiester bond and not the 2′-5′ phosphodiester bond of 2′,3′-cGAMP for cleavage. Consequently, we demonstrated for the first time that ASFV C129R and EP364R play an essential role in the cleavage of 2′,3′-cGAMP by 3′-5′ phosphodiesterase activity and inhibit subsequent 2′,3′-cGAMP-mediated type I interferon signaling.

STING is an adaptor protein that recruits and activates IKKε and TBK1, which sequentially activates the transcription factors NF-κB and IRF3, leading to the production of interferons and other cytokines. Recently, a C-terminal domain (CTD) fragment comprising residues 139 to 379 of human STING (hSTING), the sequence bound by di-GMP in bacteria, was shown to bind with natural and synthetic 2′,3′-cGAMP (11). Specifically, S161Y, Y240S, and N242A mutations in hSTING impair direct interaction with 2′,3′-cGAMP (46), and cells expressing the R232A or R232H mutant of hSTING showed defects in the secretion of IFN-β in response to 2′,3′-cGAMP and DNA (11). In addition, in the crystal structure analysis, amino acids R232, R238, V239, and Y167 of hSTING were predicted to be binding sites for 2′,3′-cGAMP (11). Notably, we found that ASFV EP364R has a region of homology with the human and porcine STING protein containing a 2′,3′-cGAMP-binding motif, and among known prospective sites, we validated the interaction between ASFV EP364R and 2′,3′-cGAMP by replacing two amino acids (Y76S and N78A) in the EP364R gene. The mutant lost the ability to form a complex with 2′,3′-cGAMP, failing to the inhibit secretion of IFN-β and IL-6 (Fig. 6). Our results suggest that tyrosine-76 and asparagine-78 in EP364R are responsible for their interaction with and degradation of 2′,3′-cGAMP and are critical for the downregulation of interferon signaling.

ASFV has adapted several proteins to control interferons and inflammatory responses. In this study, we found that two different ASFV genes had a similar function: the degradation of cGAMP. This functional redundancy raises the question of whether either of these two genes may have been lost during evolution. Conversely, these two genes are not redundant when considering the function of ASFV EP364R, which may play an essential role in repairing DNA damage and maintaining genomic stability because of its sequence homology to ERCC4 with the major Holliday junction resolvase, Mus81, from eukaryotes (6). Notably, ASFV C129R has not been identified. However, the conserved status of the EP364R N and C termini is lower than that of C129R among many different ASFV isolates. It is possible that EP364R mutations could impede phosphodiesterase activity; therefore, the functional redundancy of these two genes could be maintained as a strategy to avoid deleterious mutations of the ASFV EP364R gene.

On the other hand, multiple viral proteins targeting the same molecules/innate immune pathways and providing functional redundancy are commonly observed and have been extensively reported for large DNA viruses such as ASFV and VACV. Degradation of TBK1 by ASFV I215L (26) and ASFV MGF 360-11L (32) and NF-κB inhibition by A238L, the homolog of IκB (80), and I215L (27) are a few examples of ASFV protein functional redundancy. In contrast, ASFV I215L is involved in the virus replication cycle (81) and host protein synthesis (82), and MGF 360-11L degrades IRF7 (32), which are nonredundant functions of ASFV proteins. In vaccinia virus (VACV), C4 and C16 proteins bind to lupus Ku autoantigen protein p70 (Ku70) to antagonize DNA-PK by binding to Ku and blocking Ku binding to DNA, reducing the production of cytokines and chemokines (83). The value of coding genes with redundant functions is thought to confer increased immunomodulatory potential due to the cumulative effect of these proteins not only on one pathway but also on other cross talk pathways. Therefore, similar to VACV, ASFV appears to have evolved strategies to redundantly drive IFN and NF-κB signaling. Because of the coding capacity of ASFV and the importance of IFN and NF-κB in the antiviral response, other viral immunosuppressive proteins will probably be identified.

In summary, our study demonstrated that ASFV EP364R and C129R are negative regulators of the type I IFN signaling cascade for viral replication. To inhibit type I IFN signaling, ASFV EP364R and C129R interact directly with 2′,3′-cGAMP and mediate its cleavage, which impairs the STING self-aggregation, a key event for cGAS-STING pathway activation. These findings have important implications for the understanding of molecular mechanisms used by the ASFV EP364R and C129R to counteract the type I IFN responses and the knowledge of immune evasion strategies used by ASFV to evade host immunity. Furthermore, our study provides a rational approach for virus attenuation and ASFV vaccine development.

## MATERIALS AND METHODS

### Cells and antibodies.

HEK293T cells (ATCC CRL11268), 293-Dual hSTING-A162 cells (InvivoGen; 293d-a162), PK-15 cells (ATCC CCL-33), A549 cells (ATCC CCL-185), Vero cells (ATCC CCL-81), and MA104 cells were cultured in Dulbecco’s modified Eagle medium (DMEM) (Cytiva) and PAMs (ATCC CRL2843) in RPMI medium (Cytiva). Cells were supplemented with 10% fetal bovine serum (FBS) (Gibco) and 1% antibiotic/antimycotic (Gibco) and incubated in a humidified 5% CO_2_ incubator at 37°C. Antibodies used for the immunoblot and immunoprecipitation analysis are as follows: anti-Flag (Cell Signaling; 8146), anti-GST (Santa Cruz; sc-138), anti-Cy5 (Cell Signaling; sc-166896), anti-IRF3 (Abcam; ab25950), anti-phospho-IRF3 (Ser396) (Cell Signaling; 4947), anti-p65 (Cell Signaling; 4764S), anti-phospho-p65 (Cell Signaling; 3031S), anti-TBK1 (Cell Signaling; 3504S), anti-phospho-TBK1 (Cell Signaling; 5483S), anti-phospho-IκBα (Cell Signaling; 2859S), anti-IκBα (Cell Signaling; 9242S), anti-STING (Cell Signaling; 3337S), and anti-β-actin (Santa Cruz; SC 47778).

### Plasmids.

Plasmids with full-length ASFV C129R and EP364R genes were cloned into a Flag-tagged pIRES vector and a pEGB vector with a GST tag. To generate a Y76S N78A point mutant of EP364R-MT, a mutation cloning kit was used (Thermo Fisher; 00940669). Generation of the IFN-β promoter and luciferase reporter plasmids is described elsewhere (84). The cGAS pIRES-3×Flag-tagged plasmid was kindly donated by Jae U. Jung (Department of Cancer Biology, Lerner Research Institute, Cleveland Clinic). Flag-tagged TBK1 and IKKε plasmids were generated by amplifying template DNA using PCR and cloned into the pIRES vector. Flag-, Strep-, and GST-tagged STING plasmid constructs were obtained by amplifying template DNA by PCR and cloned into pIRES, pEXPR, and pEBG vectors, respectively. The integrity of all sequences was verified by sequencing analysis.

### Virus infection and plasmid transfection.

ADV-GFP and HSV-GFP were propagated in PK-15 cells and Vero cells, respectively, and titrated by plaque assay. Before virus infection, the culture medium was exchanged with DMEM containing 1% FBS, and the virus was inoculated into target cells at an MOI. Following 2 h of incubation at 37°C, the extracellular viruses were removed and replaced with DMEM containing 10% FBS. At the indicated times, cells were scraped with supernatants and centrifuged at 3,000 rpm for 3 min. The supernatant of each sample was separated from the cell pellet for ELISA. The cell pellet was resuspended in 300 μL of phosphate-buffered saline (PBS), and the fluorescence of each sample was checked using a fluorometer (GloMax detection system; Promega). Plasmids were transfected into PK-15 cells, PAMs, and MA104 cells with Lipofectamine 2000 (Invitrogen) and HEK293T and into 293-Dual hSTING-A162 cells with polyethylenimine (PEI; Polysciences Inc.; 23966) according to the manufacturer’s protocol.

### ASFV infection and cGAMP degradation assay.

Wild-type ASF virus (ASFV-WT) was isolated from the spleen of an infected wild boar by the National Institute of Wildlife Disease Control and Prevention (NIWDC) in Korea. This ASFV-WT strain was confirmed to be identical to the ASFV-Georgia 2007 (genotype II) by next-generation sequencing (NGS) (Celemics, South Korea). Primary porcine alveolar macrophages were provided by the National Institute of wildlife Disease Control and Prevention (NIWDC). Primary porcine cells were maintained in RPMI media and subcultured to 12-well plates. The cells were transfected with 4 μg poly(dA-dT), and at the same time, the cells were dose-dependently infected with ASFV-WT. After 2 h, the medium was changed, and the cells were incubated for a further 3 h. Then, cell supernatants and cell lysates were collected, and intracellular and extracellular cGAMP was measured by ELISA.

### Virus titer determination.

ADV-GFP- and HSV-GFP-infected cells and cell culture supernatants were collected at the indicated time points, and virus titers were measured by plaque assay using A549 and Cercopithecus aethiops epithelial kidney (Vero) cells, respectively. A monolayer of A549 and Vero cells was seeded in 12-well plates, and after 12 h of incubation, the cells were infected with serially diluted supernatants containing the virus, in 1% DMEM, for 2 h. After 2 h incubation, inoculums were removed and replaced with DMEM containing 0.1% agarose (Sigma-Aldrich). Plates were then incubated at 37°C for another 36 h and examined for plaque formation under ×200 magnification. Virus titers were calculated using the number of PFU and the dilution factor.

### ELISA.

ELISA was performed to detect secreted interferons and proinflammatory cytokines in cell culture supernatants. Human IL-6 (BD OptEIA; 5552220), human interferon-β (CUSABIO; CSB-E09889h), porcine IL-6 (R&D Systems; p6000B), and porcine IFN-β (CUSABIO; CSB-E09890p) kits were used for analysis according to the manufacturer’s protocols.

### Quantification of 2′,3′-cGAMP by ELISA.

C129R and EP364R plasmids were transfected into PK-15 cells and PAMs and infected with ADV-GFP or HSV-GFP, respectively. In another experiment, 293-Dual hSTING-A162 cells were cotransfected with C129R and EP364R plasmids with 3×Flag-cGAS plasmid. PK-15 and 293-Dual hSTING-A162 cell lysates and cell supernatants were collected and subjected to ELISA for the determination of both the intracellular and extracellular 2′,3′-cGAMP level. A 2′,3′-cGAMP ELISA (Cayman Chemical; 501700) commercial kit was used for analysis according to the manufacturer’s instructions.

### Quantitative real-time PCR.

C129R-Flag and EP364R-Flag or Flag vector as control plasmid transiently transfected PK-15 or PAMs were grown in 6-well tissue culture plates (1 × 10^6^ cells/well) and incubated at 37°C. The cells were infected with ADV-GFP (MOI = 1.0) and harvested at 0 h postinfection (hpi), 12 hpi, and 24 hpi. The total RNA from the cells was isolated using the Macherey-Nagel NucleoSpin RNA kit (790955.250), and cDNA was synthesized using reverse transcriptase (Toyobo). The different levels of cDNA were quantified by real-time PCR (RT-PCR) using a Smart Gene SYBR green Q-PCR master mix (SG.SYBR.500) kit according to the manufacturer’s instructions. The sequences of the primers used in qPCR are listed in Table S1.

### Protein immunoprecipitation.

HEK293T cells were transfected with Flag-tagged C129R, EP364R-WT, and EP364R-MT pIRES plasmids or GST-tagged C129R, EP364R-WT, and EP364R-MT pEGB plasmids and harvested at 36 hpt. The whole-cell lysates (WCL) were obtained after lysis with a protease inhibitor cocktail and phosphatase inhibitor cocktail (Sigma) containing radioimmunoprecipitation assay (RIPA) lysis buffer (50 mM Tris-HCl, 150 mM NaCl, 0.5% sodium deoxycholate, 1% IGEPAL, 1 mM NaF, 1 mM Na_3_VO_4_) and sonication with a sonicator (Sonics). The WCL was precleared with Sepharose 6B (GE Healthcare Life Science) at 4°C for 2 h. Proteins with Flag tags were precipitated using anti-Flag (M2) antibody following A/G Plus agarose (Santa Cruz Biotechnology; sc-2003) pulldown, and proteins with GST tags were precipitated with glutathione Sepharose 4B (Cytiva Sweden AB; 17075601) pulldown. Unbound proteins were washed away with lysis buffer, the fused target proteins were recovered by elution with 100 mM glycine buffer solution, pH 2 to 2.5 (Santa Cruz Biotechnology; sc-295018), and the eluted fraction was neutralized with 500 mM NH_4_HCO_3._

### Glutathione purification.

Glutathione protein was purified using the MagneGST protein purification system (Promega; V8600) with minor modifications. Briefly, GST-tagged C129R, EP364R-WT, and EP364R-MT pEGB plasmids were transfected to HEK293T cells and cells were harvested at 36 hpt. Then cells were lysed immediately with MagneGST cell lysis reagent with RNase, protease inhibitor cocktail, and phosphatase inhibitor cocktail. Next, the cell lysate was incubated with MagneGST beads at 4°C for 2 h on a rotor. Beads were separated with a magnetic separator and washed with wash buffer, followed by elution of beads by elution buffer. Afterward, the purified eluted proteins were identified by SDS-PAGE gel electrophoresis using anti-GST antibodies, and the efficiency of protein purification was evaluated by Coomassie brilliant blue staining.

### *In vitro* 2′,3′-cGAMP binding assay.

An *in vitro* binding assay was performed with minor modifications as described in a previous report (85). Briefly, 2 μg of Flag-tagged immunoprecipitated proteins or GST-purified proteins was incubated either with 10 μM 2′,3′-cGAMP-biotinylated beads (AAT Bioquest; 20316) or Cy5-conjugated 2′,3′-cGAMP (AAT Bioquest; 20318) in 10× reaction buffer containing 100 mM Tris, 500 mM KCl, 10 mM dithiothreitol (DTT), and 200 mM EDTA (pH 8) for 2 h at 37°C in the presence of the universal PDE inhibitor 1 mM IBMX (Cayman Chemical; 13347). After the incubation, the sample volume was brought up to 700 μL with RIPA buffer. To pull down the 2′,3′-cGAMP-biotinylated beads, 100 μL streptavidin magnetic beads (Dynabeads M-280 streptavidin; Thermo Fisher Scientific; 11205D) were added. To pull down the Cy5-conjugated 2′,3′-cGAMP, anti-Cy5 (Santa Cruz Biotechnology; sc-166896) monoclonal antibody was added. Then, samples were incubated for 12 h at 4°C in a rotor. For Cy5 pulldown, samples were incubated with A/G Plus agarose beads and incubated 4 h in a rotor. Beads with unbound proteins were washed with lysis buffer and incubated with 50 μL glycine elution buffer (Santa Cruz Biotechnology; sc-295018) at 100 mM and pH 2.5 for 10 min. Later, ammonium bicarbonate (500 mM) 10 μL was added for neutralization. The elution fractions were boiled in sodium dodecyl sulfate-polyacrylamide gel electrophoresis (SDS-PAGE) loading buffer (2×), and the samples were analyzed by immunoblot analysis with the indicated antibodies.

### *In vitro* 2′,3′-cGAMP degradation assay.

An *in vitro* degradation assay was performed as described previously with minor modifications (35). Degradation of 2′,3′-cGAMP was assessed by incubation of Flag-tagged immunoprecipitated protein or recombinant GST-purified protein in the presence of 2′,3′-cGAMP in a reaction buffer composed of 50 mM HEPES-KOH (pH 7.5), 35 mM KCl, and 1 mM DTT. Approximately 2 μg of total protein was normalized according to the molecular weight of each protein and incubated in a 10 μL reaction buffer with 2.5 μM 2′,3′-cGAMP with or without IBMX for 20 h at 37°C. Then the 2′,3′-cGAMP amount of each sample was measured by 2′,3′-cGAMP ELISA.

### Immunoblot analysis.

Harvested cells were lysed with RIPA lysis buffer. Cell lysates or samples prepared with immunoprecipitated beads were separated by SDS-PAGE and transferred onto a polyvinylidene difluoride (PVDF) membrane using a Trans-Blot semidry transfer cell (Bio-Rad, Seoul, South Korea). Then, the membrane was blocked for 1 h in 5% bovine serum albumin (BSA) and incubated overnight at 4°C with the primary antibody. The next day, membranes were washed with Tris-buffered saline with Tween 20 (TBST) or phosphate-buffered saline with Tween 20 (PBST), and the membrane was incubated with horseradish peroxidase (HRP)-conjugated secondary antibody for 2 h at room temperature. The membrane was washed again 3 times with TBST or PBST, and finally, the reaction was visualized using an enhanced chemiluminescence (ECL) detection system (GE Healthcare, Little Chalfont, United Kingdom) using a Las-3000 Mini Lumino image analyzer.

### Luciferase reporter assay.

HEK293T cells and 293-Dual hSTING-A162 cells were cultured in 12-well tissue culture plates (3.5 × 10^5^ cells/well) and incubated at 37°C for 12 h with a 5% CO_2_ atmosphere overnight. We used the human STING-overexpressing HEK293T cell line 293-Dual hSTING-A162 to check poly(dA-dT), cGAS- and 2′,3′-cGAMP-induced IFN-β luciferase activity, whereas HEK293T cells were used to check STING-, TBK1-, and IKKε-induced IFN-β and NF-κB luciferase reporters and poly(dA-dT), cGAS- and 2′,3′-cGAMP-induced NF-κB luciferase activity. HEK293T cells were transfected with IFN-β and NF-κB expressing luminescence, TK-Renilla luciferase reporter plasmid (an internal control for the normalization of the transfection efficiency) with Flag-tagged C129R and EP364R-WT or EP364R-MT plasmid dose dependently. Plasmids encoding cGAS, STING, TBK1, and IKKε were transfected to HEK293T cells, and 293-Dual hSTING-A162 cells were transfected with a cGAS-encoding plasmid. poly(dA-dT) (InvivoGen) and 2′,3′-cGAMP ligands (InvivoGen) were transfected with Lipofectamine 2000 (Invitrogen) and Lipofectamine RNAiMAX (Invitrogen), respectively. At 24 h posttransfection, luciferase activity was measured using a dual-luciferase reporter assay system (Promega; E1980) following the manufacturer’s protocol.

### 2′,3′-cGAMP secretion and conditioned-medium transfer experiment.

PAMs were infected with HSV-GFP (MOI = 0.5, 1, 3, and 5) and ADV-GFP (MOI = 1, 2, and 4) for 4 h, and collected supernatants were assessed for the 2′,3′-cGAMP secretion using ELISA. Next, PAMs were cotransfected with either Flag-tagged EP364R or C129R dose dependently and then infected with HSV-GFP (MOI = 5) and ADV-GFP (MOI = 4) for 4 h, and 2′,3′-cGAMP secretion was assessed by ELISA. Then, 293-Dual hSTING-A162 cells were cotransfected with Flag-EP364R or Flag-C129R dose dependently with cGAS plasmid with a Flag vector as the transfection control. At 24 hpt, the cell supernatant was collected, and media were transferred to a monolayer of PAMs and incubated for 24 h. Finally, IFN-β transcription and intracellular 2′,3′-cGAMP level in PAMs were measured compared to untreated control cells by ELISA. The remaining supernatant of 293-Dual hSTING-A162 cells was used to assess the secretion of 2′,3′-cGAMP by ELISA.

### Phosphodiesterase activity assay.

PK-15 cells were transfected with Flag-EP364R and Flag-C129R, and then cells were infected with ADV-GFP (MOI = 1) and treated with 200 nM IBMX at 6 hpt. GFP microscopy and GFP absorbance, virus replication, IFN-β secretion, and IL-6 secretion were measured at 12 h and 24 h. Next, 293-Dual hSTING-A162 cells were cotransfected with Flag-cGAS plasmid with Flag-C129R or EP364R plasmids dose dependently, and 6 h before cells were harvested, they were treated with 100 nM IBMX. Then, cells and supernatants were harvested 24 hpt to check intracellular and extracellular 2′,3′-cGAMP levels. Finally, an *in vitro* 2′,3′-cGAMP degradation assay determined the degradation of 2′,3′-cGAMP with or without IBMX. Immunoprecipitated and purified GST-tagged EP364R and C129R proteins plus 2.5 μM 2′,3′-cGAMP with 1 mM IBMX were incubated 22 h at 37°C in the reaction buffer followed by 2′,3′-cGAMP ELISA to quantify the remaining 2′,3′-cGAMP level in incubated samples.

### HPLC analysis.

ASFV genes expressing Flag-tagged C129R, EP364R-WT, and EP364R-MT or Flag control plasmids were transfected with 3×Flag cGAS plasmid into HEK293T cells. Then, cells were harvested at 36 hpt. Cellular nucleotide extraction was performed as previously described (86). Harvested cells were resuspended in 300 μL of ice-cold extraction solvent containing acetonitrile-methanol-water (40/40/20 [vol/vol/vol]). The extraction process was initiated after 15 min of incubation at 4°C. The cell suspension was then heated to 95°C for 10 min. The cell suspension was cooled and centrifuged at 20,000 × *g* for 5 min, and the insoluble fraction was separated from the extracted nucleotides. The remaining cell pellet was extracted twice with 200 μL of extraction solvent at 4°C without a heating step. The solvent of the combined supernatants (700 μL) was then evaporated until dry in a vacuum evaporator (Modul 3180C; Hanil Research and Development). The dried fraction was resuspended in 200 μL of ultrapure water with vigorous vortexing and then analyzed by reversed-phase-coupled HPLC-tandem mass spectrometry (MS/MS).

HPLC analysis was performed following the protocol described previously (36). Extracted nucleotides were analyzed via LC-MS/MS on a QTRAP 6500 low-mass BL210251506 instrument using an electrospray ionization probe and a Shimadzu Prominence UFLC XR UHPLC system. A reverse-phase C_18_ column (2.1 mm by 50 mm, 3.5-μm particles, 125-Å pores) was used. The mobile phases consisted of 0.1% formic acid (A) in water and 0.1% formic acid (B) in acetonitrile at a flow rate of 0.3 mL/min. The run was composed of a linear gradient of 0 to 100% B over 12 min, 100% B for 3 min, and then 0% B for 4 min. MS was conducted in negative-ion multiple reaction monitoring (MRM) mode. For 2′,3′-cGAMP, the [M-H] precursor ion of *m/z* 673.1 and production of *m/z* 344.1 (quantifier) and 328.1 (qualifier) for GMP *m/z* 362.0/211 and AMP *m/z* 345.9/211.0 were used.

### SDD-AGE assay.

293-Dual hSTING-A162 cells were transfected with Flag-tagged C129R, EP364R, and its mutant dose dependently with 3×Flag-tagged cGAS plasmids. Twenty-four hours later, 8 μg of synthesized 2′,3′-cGAMP ligand was transfected with RNAiMAX, and then cells were harvested at 12 hpt. Then, cells were washed with PBS and resuspended with lysis buffer. Cell fractions were separated by centrifugation, and the supernatant was mixed with SDS-AGE buffer and separated by 1.5% SDD-AGE as previously described (48).

### Statistical analysis.

Graphs and all statistical analyses were performed using GraphPad Prism software version 6 for Windows. Data are presented as the means and standard deviations (SD) and represent at least two independent experiments. An unpaired *t* test was performed to compare the control and treatment groups at each time point. *P* values of <0.05, <0.01, <0.001 or <0.0001 were regarded as significant.

## References

[1] Gaudreault NN, Madden DW, Wilson WC, Trujillo JD, Richt JA. 2020. African swine fever virus: an emerging DNA arbovirus. Front Vet Sci 7:215. 10.3389/fvets.2020.00215. 32478103 PMC7237725

[2] Alonso C, Borca M, Dixon L, Revilla Y, Rodriguez F, Escribano J, ICTV Report Committee. 2018. ICTV virus taxonomy profile: Asfarviridae. J Gen Virol 99:613–614. 10.1099/jgv.0.001049. 29565243 PMC12662184

[3] Xia N, Wang H, Liu X, Shao Q, Ao D, Xu Y, Jiang S, Luo J, Zhang J, Chen N, Meurens F, Zheng W, Zhu J. 2020. African swine fever virus structural protein p17 inhibits cell proliferation through ER stress—ROS mediated cell cycle arrest. Viruses 13:21. 10.3390/v13010021. PMC7823474 33374251

[4] Alejo A, Matamoros T, Guerra M, Andrés G. 2018. A proteomic atlas of the African swine fever virus particle. J Virol 92:e01293-18. 10.1128/JVI.01293-18. 30185597 PMC6232493

[5] Karger A, Pérez-Núñez D, Urquiza J, Hinojar P, Alonso C, Freitas FB, Revilla Y, Le Potier M-F, Montoya M. 2019. An update on African swine fever virology. Viruses 11:864. 10.3390/v11090864. PMC6784044 31533244

[6] Dixon LK, Chapman DA, Netherton CL, Upton C. 2013. African swine fever virus replication and genomics. Virus Res 173:3–14. 10.1016/j.virusres.2012.10.020. 23142553

[7] Aicher S-M, Monaghan P, Netherton CL, Hawes PC. 2021. Unpicking the secrets of African swine fever viral replication sites. Viruses 13:77. 10.3390/v13010077. 33429879 PMC7827680

[8] Dixon L, Sun H, Roberts H. 2019. African swine fever. Antiviral Res 165:34–41. 10.1016/j.antiviral.2019.02.018. 30836106

[9] Galindo I, Alonso C. 2017. African swine fever virus: a review. Viruses 9:103. 10.3390/v9050103. PMC5454416 28489063

[10] Kato K, Omura H, Ishitani R, Nureki O. 2017. Cyclic GMP-AMP as an endogenous second messenger in innate immune signaling by cytosolic DNA. Annu Rev Biochem 86:541–566. 10.1146/annurev-biochem-061516-044813. 28399655

[11] Zhang X, Shi H, Wu J, Zhang X, Sun L, Chen C, Chen ZJ. 2013. Cyclic GMP-AMP containing mixed phosphodiester linkages is an endogenous high-affinity ligand for STING. Mol Cell 51:226–235. 10.1016/j.molcel.2013.05.022. 23747010 PMC3808999

[12] Ablasser A, Goldeck M, Cavlar T, Deimling T, Witte G, Röhl I, Hopfner KP, Ludwig J, Hornung V. 2013. cGAS produces a 2'-5'-linked cyclic dinucleotide second messenger that activates STING. Nature 498:380–384. 10.1038/nature12306. 23722158 PMC4143541

[13] Diner EJ, Burdette DL, Wilson SC, Monroe KM, Kellenberger CA, Hyodo M, Hayakawa Y, Hammond MC, Vance RE. 2013. The innate immune DNA sensor cGAS produces a noncanonical cyclic dinucleotide that activates human STING. Cell Rep 3:1355–1361. 10.1016/j.celrep.2013.05.009. 23707065 PMC3706192

[14] Zhong B, Yang Y, Li S, Wang YY, Li Y, Diao F, Lei C, He X, Zhang L, Tien P, Shu HB. 2008. The adaptor protein MITA links virus-sensing receptors to IRF3 transcription factor activation. Immunity 29:538–550. 10.1016/j.immuni.2008.09.003. 18818105

[15] Tanaka Y, Chen ZJ. 2012. STING specifies IRF3 phosphorylation by TBK1 in the cytosolic DNA signaling pathway. Sci Signal 5:ra20. 10.1126/scisignal.2002521. 22394562 PMC3549669

[16] Fitzgerald KA, McWhirter SM, Faia KL, Rowe DC, Latz E, Golenbock DT, Coyle AJ, Liao SM, Maniatis T. 2003. IKKepsilon and TBK1 are essential components of the IRF3 signaling pathway. Nat Immunol 4:491–496. 10.1038/ni921. 12692549

[17] Sharma S, tenOever BR, Grandvaux N, Zhou GP, Lin R, Hiscott J. 2003. Triggering the interferon antiviral response through an IKK-related pathway. Science 300:1148–1151. 10.1126/science.1081315. 12702806

[18] Dixon LK, Islam M, Nash R, Reis AL. 2019. African swine fever virus evasion of host defences. Virus Res 266:25–33. 10.1016/j.virusres.2019.04.002. 30959069 PMC6505686

[19] Ma Z, Damania B. 2016. The cGAS-STING defense pathway and its counteraction by viruses. Cell Host Microbe 19:150–158. 10.1016/j.chom.2016.01.010. 26867174 PMC4755325

[20] García-Belmonte R, Pérez-Núñez D, Pittau M, Richt JA, Revilla Y. 2019. African swine fever virus Armenia/07 virulent strain controls interferon beta production through the cGAS-STING pathway. J Virol 93:e02298-18. 10.1128/JVI.02298-18. 30918080 PMC6613762

[21] Fan W, Jiao P, Zhang H, Chen T, Zhou X, Qi Y, Sun L, Shang Y, Zhu H, Hu R, Liu W, Li J. 2020. Inhibition of African swine fever virus replication by porcine type I and type II interferons. Front Microbiol 11:1203. 10.3389/fmicb.2020.01203. 32655518 PMC7325991

[22] Li D, Yang W, Li L, Li P, Ma Z, Zhang J, Qi X, Ren J, Ru Y, Niu Q, Liu Z, Liu X, Zheng H. 2021. African swine fever virus MGF-505-7R negatively regulates cGAS–STING-mediated signaling pathway. J Immunol 206:1844–1857. 10.4049/jimmunol.2001110. 33712518 PMC8023146

[23] Li J, Song J, Kang L, Huang L, Zhou S, Hu L, Zheng J, Li C, Zhang X, He X, Zhao D, Bu Z, Weng C. 2021. pMGF505-7R determines pathogenicity of African swine fever virus infection by inhibiting IL-1β and type I IFN production. PLoS Pathog 17:e1009733. 10.1371/journal.ppat.1009733. 34310655 PMC8341718

[24] Wang X, Wu J, Wu Y, Chen H, Zhang S, Li J, Xin T, Jia H, Hou S, Jiang Y, Zhu H, Guo X. 2018. Inhibition of cGAS-STING-TBK1 signaling pathway by DP96R of ASFV China 2018/1. Biochem Biophys Res Commun 506:437–443. 10.1016/j.bbrc.2018.10.103. 30348523

[25] Liu H, Zhu Z, Feng T, Ma Z, Xue Q, Wu P, Li P, Li S, Yang F, Cao W, Xue Z, Chen H, Liu X, Zheng H. 2021. African swine fever virus E120R protein inhibits interferon-β production by interacting with IRF3 to block its activation. J Virol 95:e00824-21. 10.1128/JVI.00824-21. PMC8387055 34190598

[26] Huang L, Xu W, Liu H, Xue M, Liu X, Zhang K, Hu L, Li J, Liu X, Xiang Z, Zheng J, Li C, Chen W, Bu Z, Xiong T, Weng C. 2021. African swine fever virus pI215L negatively regulates cGAS-STING signaling pathway through recruiting RNF138 to inhibit K63-linked ubiquitination of TBK1. J Immunol 207:2754–2769. 10.4049/jimmunol.2100320. 34759016

[27] Barrado-Gil L, del Puerto A, Galindo I, Cuesta-Geijo MÁ, García-Dorival I, de Motes CM, Alonso C. 2021. African swine fever virus ubiquitin-conjugating enzyme is an immunomodulator targeting NF-κB activation. Viruses 13:1160. 10.3390/v13061160. 34204411 PMC8233900

[28] Ran Y, Li D, Xiong MG, Liu HN, Feng T, Shi ZW, Li YH, Wu HN, Wang SY, Zheng HX, Wang YY. 2022. African swine fever virus I267L acts as an important virulence factor by inhibiting RNA polymerase III-RIG-I-mediated innate immunity. PLoS Pathog 18:e1010270. 10.1371/journal.ppat.1010270. 35089988 PMC8827485

[29] Yang J, Li S, Feng T, Zhang X, Yang F, Cao W, Chen H, Liu H, Zhang K, Zhu Z, Zheng H. 2021. African swine fever virus F317L protein inhibits NF-κB activation to evade host immune response and promote viral replication. mSphere 6:e00658-21. 10.1128/mSphere.00658-21. PMC8527992 34668754

[30] Zhuo Y, Guo Z, Ba T, Zhang C, He L, Zeng C, Dai H. 2021. African swine fever virus MGF360-12L inhibits type I interferon production by blocking the interaction of importin α and NF-κB signaling pathway. Virol Sin 36:176–186. 10.1007/s12250-020-00304-4. 33141406 PMC7606853

[31] Yang K, Huang Q, Wang R, Zeng Y, Cheng M, Xue Y, Shi C, Ye L, Yang W, Jiang Y, Wang J, Huang H, Cao X, Yang G, Wang C. 2021. African swine fever virus MGF505-11R inhibits type I interferon production by negatively regulating the cGAS-STING-mediated signaling pathway. Vet Microbiol 263:109265. 10.1016/j.vetmic.2021.109265. 34710767

[32] Yang K, Xue Y, Niu H, Shi C, Cheng M, Wang J, Zou B, Wang J, Niu T, Bao M, Yang W, Zhao D, Jiang Y, Yang G, Zeng Y, Cao X, Wang C. 2022. African swine fever virus MGF360-11L negatively regulates cGAS-STING-mediated inhibition of type I interferon production. Vet Res 53:7–12. 10.1186/s13567-022-01025-0. 35073979 PMC8785597

[33] Holly MK, Smith JG. 2018. Adenovirus infection of human enteroids reveals interferon sensitivity and preferential infection of goblet cells. J Virol 92:e00250-18. 10.1128/JVI.00250-18. 29467318 PMC5899204

[34] Altinkilic B, Brandner G. 1988. Interferon inhibits herpes simplex virus-specific translation: a reinvestigation. J Gen Virol 69:3107–3112. 10.1099/0022-1317-69-12-3107. 2848929

[35] Eaglesham JB, Pan Y, Kupper TS, Kranzusch PJ. 2019. Viral and metazoan poxins are cGAMP-specific nucleases that restrict cGAS–STING signalling. Nature 566:259–263. 10.1038/s41586-019-0928-6. 30728498 PMC6640140

[36] Dey RJ, Dey B, Zheng Y, Cheung LS, Zhou J, Sayre D, Kumar P, Guo H, Lamichhane G, Sintim HO, Bishai WR. 2017. Inhibition of innate immune cytosolic surveillance by an M. tuberculosis phosphodiesterase. Nat Chem Biol 13:210–217. 10.1038/nchembio.2254. 28106876

[37] Li L, Yin Q, Kuss P, Maliga Z, Millán JL, Wu H, Mitchison TJ. 2014. Hydrolysis of 2′3′-cGAMP by ENPP1 and design of nonhydrolyzable analogs. Nat Chem Biol 10:1043–1048. 10.1038/nchembio.1661. 25344812 PMC4232468

[38] Zhou C, Chen X, Planells-Cases R, Chu J, Wang L, Cao L, Li Z, López-Cayuqueo KI, Xie Y, Ye S, Wang X, Ullrich F, Ma S, Fang Y, Zhang X, Qian Z, Liang X, Cai S-Q, Jiang Z, Zhou D, Leng Q, Xiao TS, Lan K, Yang J, Li H, Peng C, Qiu Z, Jentsch TJ, Xiao H. 2020. Transfer of cGAMP into bystander cells via LRRC8 volume-regulated anion channels augments STING-mediated interferon responses and anti-viral immunity. Immunity 52:767–781.E6. 10.1016/j.immuni.2020.03.016. 32277911

[39] Carozza JA, Böhnert V, Nguyen KC, Skariah G, Shaw KE, Brown JA, Rafat M, von Eyben R, Graves EE, Glenn JS, Smith M, Li L. 2020. Extracellular cGAMP is a cancer-cell-produced immunotransmitter involved in radiation-induced anticancer immunity. Nat Cancer 1:184–196. 10.1038/s43018-020-0028-4. 33768207 PMC7990037

[40] Kato K, Nishimasu H, Oikawa D, Hirano S, Hirano H, Kasuya G, Ishitani R, Tokunaga F, Nureki O. 2018. Structural insights into cGAMP degradation by Ecto-nucleotide pyrophosphatase phosphodiesterase 1. Nat Commun 9:4424. 10.1038/s41467-018-06922-7. 30356045 PMC6200793

[41] Deng MJ, Tao J, Chao E, Ye ZY, Jiang Z, Yu J, Su XD. 2018. Novel mechanism for cyclic dinucleotide degradation revealed by structural studies of Vibrio phosphodiesterase V-cGAP3. J Mol Biol 430:5080–5093. 10.1016/j.jmb.2018.10.010. 30365951

[42] Latoscha A, Drexler DJ, Al-Bassam MM, Bandera AM, Kaever V, Findlay KC, Witte G, Tschowri N. 2020. c-di-AMP hydrolysis by the phosphodiesterase AtaC promotes differentiation of multicellular bacteria. Proc Natl Acad Sci USA 117:7392–7400. 10.1073/pnas.1917080117. 32188788 PMC7132281

[43] Yadav M, Pal K, Sen U. 2019. Structures of c-di-GMP/cGAMP degrading phosphodiesterase Vc EAL: identification of a novel conformational switch and its implication. Biochem J 476:3333–3353. 10.1042/BCJ20190399. 31647518

[44] Hernáez B, Alonso G, Georgana I, El-Jesr M, Martín R, Shair KH, Fischer C, Sauer S, De Motes CM, Alcamí A. 2020. Viral cGAMP nuclease reveals the essential role of DNA sensing in protection against acute lethal virus infection. Sci Adv 6:eabb4565. 10.1126/sciadv.abb4565. 32948585 PMC7500930

[45] Eaglesham JB, McCarty KL, Kranzusch PJ. 2020. Structures of diverse poxin cGAMP nucleases reveal a widespread role for cGAS-STING evasion in host–pathogen conflict. Elife 9:e59753. 10.7554/eLife.59753. 33191912 PMC7688311

[46] Wu J, Sun L, Chen X, Du F, Shi H, Chen C, Chen ZJ. 2013. Cyclic GMP-AMP is an endogenous second messenger in innate immune signaling by cytosolic DNA. Science 339:826–830. 10.1126/science.1229963. 23258412 PMC3855410

[47] He Y, Hong C, Yan EZ, Fletcher SJ, Zhu G, Yang M, Li Y, Sun X, Irvine DJ, Li J, Hammond PT. 2020. Self-assembled cGAMP-STINGΔTM signaling complex as a bioinspired platform for cGAMP delivery. Sci Adv 6:eaba7589. 10.1126/sciadv.aba7589. 32582856 PMC7292616

[48] Wang P-H, Fung S-Y, Gao W-W, Deng J-J, Cheng Y, Chaudhary V, Yuen K-S, Ho T-H, Chan C-P, Zhang Y, Kok K-H, Yang W, Chan C-P, Jin D-Y. 2018. A novel transcript isoform of STING that sequesters cGAMP and dominantly inhibits innate nucleic acid sensing. Nucleic Acids Res 46:4054–4071. 10.1093/nar/gky186. 29547894 PMC5934658

[49] Lee H-C, Chathuranga K, Lee J-S. 2019. Intracellular sensing of viral genomes and viral evasion. Exp Mol Med 51:1–13. 10.1038/s12276-019-0299-y. PMC6906418 31827068

[50] Gui X, Yang H, Li T, Tan X, Shi P, Li M, Du F, Chen ZJ. 2019. Autophagy induction via STING trafficking is a primordial function of the cGAS pathway. Nature 567:262–266. 10.1038/s41586-019-1006-9. 30842662 PMC9417302

[51] Fryer AL, Abdullah A, Taylor JM, Crack PJ. 2021. The complexity of the cGAS-STING pathway in CNS pathologies. Front Neurosci 15:621501. 10.3389/fnins.2021.621501. 33633536 PMC7900568

[52] Motwani M, Pesiridis S, Fitzgerald KA. 2019. DNA sensing by the cGAS–STING pathway in health and disease. Nat Rev Genet 20:657–674. 10.1038/s41576-019-0151-1. 31358977

[53] Zheng J, Mo J, Zhu T, Zhuo W, Yi Y, Hu S, Yin J, Zhang W, Zhou H, Liu Z. 2020. Comprehensive elaboration of the cGAS-STING signaling axis in cancer development and immunotherapy. Mol Cancer 19:1–19. 10.1186/s12943-020-01250-1. 32854711 PMC7450153

[54] Cheng Z, Dai T, He X, Zhang Z, Xie F, Wang S, Zhang L, Zhou F. 2020. The interactions between cGAS-STING pathway and pathogens. Sig Transduct Target Ther 5:1–15. 10.1038/s41392-020-0198-7. PMC7293265 32532954

[55] Vavřina Z, Gutten O, Smola M, Zavřel M, Aliakbar Tehrani Z, Charvát V, Kožíšek M, Boura E, Birkuš G, Rulíšek L. 2021. Protein-ligand interactions in the STING binding site probed by rationally designed single-point mutations: experiment and theory. Biochemistry 60:607–620. 10.1021/acs.biochem.0c00949. 33586948

[56] Danilchanka O, Mekalanos JJ. 2013. Cyclic dinucleotides and the innate immune response. Cell 154:962–970. 10.1016/j.cell.2013.08.014. 23993090 PMC3931520

[57] Römling U, Gomelsky M, Galperin MY. 2005. C‐di‐GMP: the dawning of a novel bacterial signalling system. Mol Microbiol 57:629–639. 10.1111/j.1365-2958.2005.04697.x. 16045609

[58] Gentili M, Kowal J, Tkach M, Satoh T, Lahaye X, Conrad C, Boyron M, Lombard B, Durand S, Kroemer G, Loew D, Dalod M, Théry C, Manel N. 2015. Transmission of innate immune signaling by packaging of cGAMP in viral particles. Science 349:1232–1236. 10.1126/science.aab3628. 26229115

[59] Dialer CR, Stazzoni S, Drexler DJ, Müller FM, Veth S, Pichler A, Okamura H, Witte G, Hopfner K-P, Carell T. 2019. A click-chemistry linked 2’3’-cGAMP analog. Chem Eur J 25:2089–2095. 10.1002/chem.201805409. 30536650

[60] Gao P, Ascano M, Zillinger T, Wang W, Dai P, Serganov AA, Gaffney BL, Shuman S, Jones RA, Deng L, Hartmann G, Barchet W, Tuschl T, Patel DJ. 2013. Structure-function analysis of STING activation by c[G(2′,5′)pA (3′,5′)p] and targeting by antiviral DMXAA. Cell 154:748–762. 10.1016/j.cell.2013.07.023. 23910378 PMC4386733

[61] Lim J, Kim H-Y. 2020. Novel applications of biocatalysis to stereochemistry determination of 2′3′-cGAMP bisphosphorothioate (2′3′-cGSASMP). ACS Omega 5:14173–14179. 10.1021/acsomega.0c01942. 32566885 PMC7301576

[62] Wang C, Hao M, Qi Q, Chen Y, Hartig JS. 2019. Chemical synthesis, purification, and characterization of 3′-5′-linked canonical cyclic dinucleotides (CDNs). Methods Enzymol 625:41–59. 10.1016/bs.mie.2019.04.022. 31455536

[63] Ablasser A, Schmid-Burgk JL, Hemmerling I, Horvath GL, Schmidt T, Latz E, Hornung V. 2013. Cell intrinsic immunity spreads to bystander cells via the intercellular transfer of cGAMP. Nature 503:530–534. 10.1038/nature12640. 24077100 PMC4142317

[64] Chen Q, Boire A, Jin X, Valiente M, Er EE, Lopez-Soto A, Jacob L, Patwa R, Shah H, Xu K, Cross JR, Massagué J. 2016. Carcinoma–astrocyte gap junctions promote brain metastasis by cGAMP transfer. Nature 533:493–498. 10.1038/nature18268. 27225120 PMC5021195

[65] Marcus A, Mao AJ, Lensink-Vasan M, Wang L, Vance RE, Raulet DH. 2018. Tumor-derived cGAMP triggers a STING-mediated interferon response in non-tumor cells to activate the NK cell response. Immunity 49:754–763.E4. 10.1016/j.immuni.2018.09.016. 30332631 PMC6488306

[66] Bridgeman A, Maelfait J, Davenne T, Partridge T, Peng Y, Mayer A, Dong T, Kaever V, Borrow P, Rehwinkel J. 2015. Viruses transfer the antiviral second messenger cGAMP between cells. Science 349:1228–1232. 10.1126/science.aab3632. 26229117 PMC4617605

[67] Liu D, Wu H, Wang C, Li Y, Tian H, Siraj S, Sehgal SA, Wang X, Wang J, Shang Y, Jiang Z, Liu L, Chen Q. 2019. STING directly activates autophagy to tune the innate immune response. Cell Death Differ 26:1735–1749. 10.1038/s41418-018-0251-z. 30568238 PMC6748081

[68] Wang W, Hu D, Wu C, Feng Y, Li A, Liu W, Wang Y, Chen K, Tian M, Xiao F, Zhang Q, Shereen MA, Chen W, Pan P, Wan P, Wu K, Wu J. 2020. STING promotes NLRP3 localization in ER and facilitates NLRP3 deubiquitination to activate the inflammasome upon HSV-1 infection. PLoS Pathog 16:e1008335. 10.1371/journal.ppat.1008335. 32187211 PMC7080238

[69] Eaglesham JB, Kranzusch PJ. 2020. Conserved strategies for pathogen evasion of cGAS–STING immunity. Curr Opin Immunol 66:27–34. 10.1016/j.coi.2020.04.002. 32339908 PMC7158794

[70] Zhao L, Jha BK, Wu A, Elliott R, Ziebuhr J, Gorbalenya AE, Silverman RH, Weiss SR. 2012. Antagonism of the interferon-induced OAS-RNase L pathway by murine coronavirus ns2 protein is required for virus replication and liver pathology. Cell Host Microbe 11:607–616. 10.1016/j.chom.2012.04.011. 22704621 PMC3377938

[71] Zhang R, Jha BK, Ogden KM, Dong B, Zhao L, Elliott R, Patton JT, Silverman RH, Weiss SR. 2013. Homologous 2′,5′-phosphodiesterases from disparate RNA viruses antagonize antiviral innate immunity. Proc Natl Acad Sci USA 110:13114–13119. 10.1073/pnas.1306917110. 23878220 PMC3740845

[72] Golub EE. 2009. Role of matrix vesicles in biomineralization. Biochim Biophys Acta 1790:1592–1598. 10.1016/j.bbagen.2009.09.006. 19786074 PMC2783689

[73] Evans WH, Hood DO, Gurd JW. 1973. Purification and properties of a mouse liver plasma-membrane glycoprotein hydrolysing nucleotide pyrophosphate and phosphodiester bonds. Biochem J 135:819–826. 10.1042/bj1350819. 4360250 PMC1165900

[74] Yang W. 2011. Nucleases: diversity of structure, function and mechanism. Q Rev Biophys 44:1–93. 10.1017/S0033583510000181. 20854710 PMC6320257

[75] Maurice DH, Ke H, Ahmad F, Wang Y, Chung J, Manganiello VC. 2014. Advances in targeting cyclic nucleotide phosphodiesterases. Nat Rev Drug Discov 13:290–314. 10.1038/nrd4228. 24687066 PMC4155750

[76] Baillie GS, Tejeda GS, Kelly MP. 2019. Therapeutic targeting of 3′,5′-cyclic nucleotide phosphodiesterases: inhibition and beyond. Nat Rev Drug Discov 18:770–796. 10.1038/s41573-019-0033-4. 31388135 PMC6773486

[77] Fawcett L, Baxendale R, Stacey P, McGrouther C, Harrow I, Soderling S, Hetman J, Beavo JA, Phillips SC. 2000. Molecular cloning and characterization of a distinct human phosphodiesterase gene family: PDE11A. Proc Natl Acad Sci USA 97:3702–3707. 10.1073/pnas.97.7.3702. 10725373 PMC16303

[78] Gassler J, Flyamer IM, Tachibana K. 2018. Single-nucleus Hi-C of mammalian oocytes and zygotes. Methods Cell Biol 144:389–407. 10.1016/bs.mcb.2018.03.032. 29804679

[79] Ahmad F, Murata T, Shimizu K, Degerman E, Maurice D, Manganiello V. 2015. Cyclic nucleotide phosphodiesterases: important signaling modulators and therapeutic targets. Oral Dis 21:e25–e50. 10.1111/odi.12275. 25056711 PMC4275405

[80] Revilla Y, Callejo M, Rodríguez JM, Culebras E, Nogal ML, Salas ML, Viñuela E, Fresno M. 1998. Inhibition of nuclear factor κB activation by a virus-encoded IκB-like protein. J Biol Chem 273:5405–5411. 10.1074/jbc.273.9.5405. 9479002

[81] Freitas FB, Frouco G, Martins C, Ferreira F. 2018. African swine fever virus encodes for an E2-ubiquitin conjugating enzyme that is mono-and di-ubiquitinated and required for viral replication cycle. Sci Rep 8:3471. 10.1038/s41598-018-21872-2. 29472632 PMC5823848

[82] Barrado-Gil L, Del Puerto A, Muñoz-Moreno R, Galindo I, Cuesta-Geijo MÁ, Urquiza J, Nistal-Villán E, Maluquer de Motes C, Alonso C. 2020. African swine fever virus ubiquitin-conjugating enzyme interacts with host translation machinery to regulate the host protein synthesis. Front Microbiol 11:3186. 10.3389/fmicb.2020.622907. PMC7771050 33384682

[83] Scutts SR, Ember SW, Ren H, Ye C, Lovejoy CA, Mazzon M, Veyer DL, Sumner RP, Smith GL. 2018. DNA-PK is targeted by multiple vaccinia virus proteins to inhibit DNA sensing. Cell Rep 25:1953–1965.E4. 10.1016/j.celrep.2018.10.034. 30428360 PMC6250978

[84] Kim J-H, Kim T-H, Lee H-C, Nikapitiya C, Uddin MB, Park M-E, Pathinayake P, Lee ES, Chathuranga K, Herath TUB, Chathuranga WAG, Lee J-S. 2017. Rubicon modulates antiviral type I interferon (IFN) signaling by targeting IFN regulatory factor 3 dimerization. J Virol 91:e00248-17. 10.1128/JVI.00248-17. 28468885 PMC5487567

[85] Chambers JR, Sauer K. 2017. Detection of cyclic di-GMP binding proteins utilizing a biotinylated cyclic di-GMP pulldown assay. Methods Mol Biol 1657:317–329. 10.1007/978-1-4939-7240-1_25. 28889305 PMC5702493

[86] Spangler C, Böhm A, Jenal U, Seifert R, Kaever V. 2010. A liquid chromatography-coupled tandem mass spectrometry method for quantitation of cyclic di-guanosine monophosphate. J Microbiol Methods 81:226–231. 10.1016/j.mimet.2010.03.020. 20385176

