# Supplementary material for: African Swine Fever Virus EP364R and C129R Target Cyclic GMP-AMP To Inhibit the cGAS-STING Signaling Pathway
Source: J Virol. 2022 Jul 21;96(15):e01022-22. doi: 10.1128/jvi.01022-22 (PMC9364804; doi:10.1128/jvi.01022-22)
Supplement: Supplemental file 1 — Fig. S1 to S7 and Table S1. Download jvi.01022-22-s0001.pdf, PDF file, 1.7 MB [file jvi.01022-22-s0001.pdf]

TABLE S1: List of primers used for real time PCR

Target gene	Forward primer	Reverse primer
pIFN-β	AAATCGCTCTCTGATGTGT	TGCTCCTTTGTTGGTATCG
pIFN-γ	CCATTCAAAGGAGCATGGAT	ATCCATGCTCCTTTGAATGG
pIL-6	CACCGGTCTTGTGGAGTTTC	GTGGTGGCTTTGTCTGGATT
pIL-1β	GGGACTTGAAGAGAGAAGTGG	CTTTCCCTTGATCCCTAAGGT
pTNFα	TCACAGGGCAATGATCCC	GGGATCATTGCCCTGTGA
pMCP-1	CAGAAGAGTCACCAGCAGCA	TCCAGGTGGCTTATGGAGTC
pMX-1	TAGGCAATCAGCCATACG	GTTGATGGTCTCCTGCTTAC
pISG-15	AAATCGCTCTCCTGATGTGT	TGCTCCTTTGTTGGTATCG

Supplementary figures

FIG S1

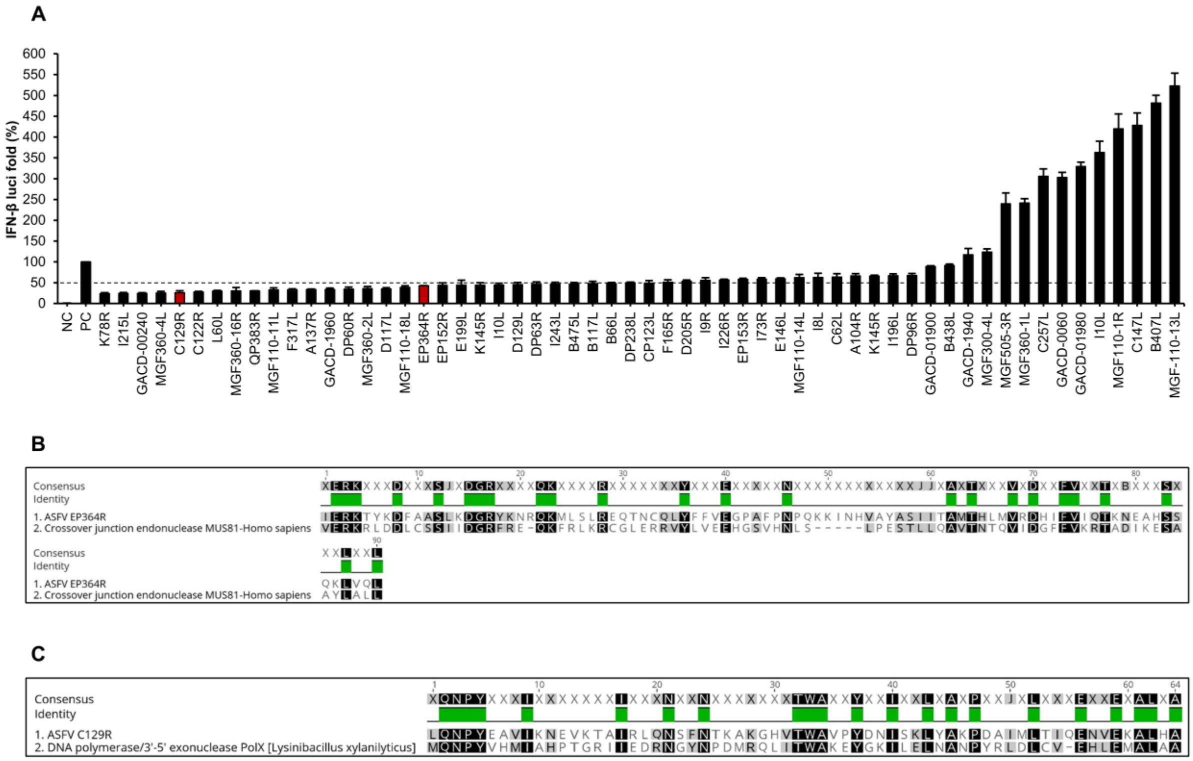

FIG S1 Screening results of ASFV genes library

(A) 293-Dual™ hSTING-A162 cells were transfected with indicated individual ASFV gene for 24 h. Then 4 μg of 2’3’cGAMP ligand was transfected into the cells and IFN-β luciferase activity was measured at 12 h post treatment. (B) Sequence alignment of ASFV EP364R with crossover junction endonuclease MUS81 (*Homo sapiens*) and (C) sequence alignment of ASFV C129R with DNA polymerase/ 3’-5’ exonuclease PolX (*Lysinibacillus xylanilyticus*).

FIG S2

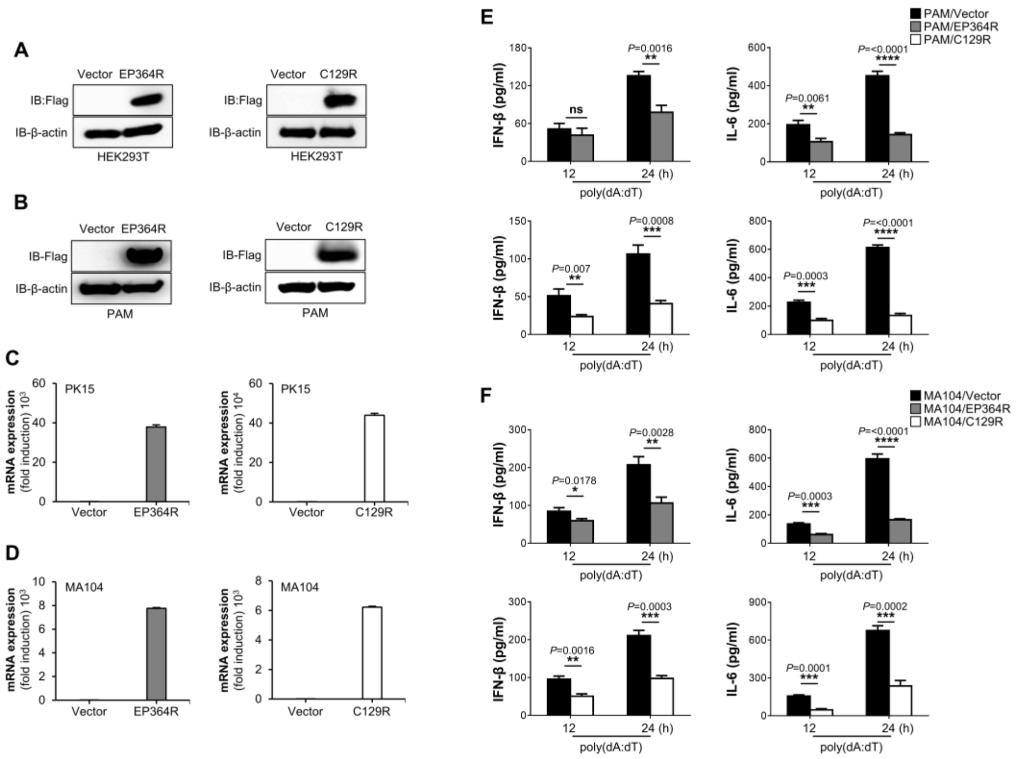

FIG S2 Overexpression of EP364R and C129R suppresses the IFN and cytokine production

Protein expression of overexpressed EP364R and C129R in HEK293T (A) and PAM cells (B). Gene transcription level of EP364R and C129R in PK-15 (C), and MA104 cells (D) Cells were transfected with an IRES vector or Flag-EP364R or Flag-C129R and cDNA was synthesized, followed by qRT-PCR. PAM cells (E) and MA104 cells (F) were transfected with Flag-EP364R and Flag-C129R plasmids and treated with poly(dA:dT) at 24 hpt. At the indicated time points, cell supernatants were collected and IFN-β and IL-6 secretions were measured. All the data are representative of at least two independent experiments, each with similar results, and the values are expressed as mean ± SD of three biological replicates. All the immunoblot data are

representative of at least two independent experiments, each with similar results. Student's t-test; * $p < 0.05$; ** $p < 0.01$; *** $p < 0.001$; ns, not significant.

FIG S3

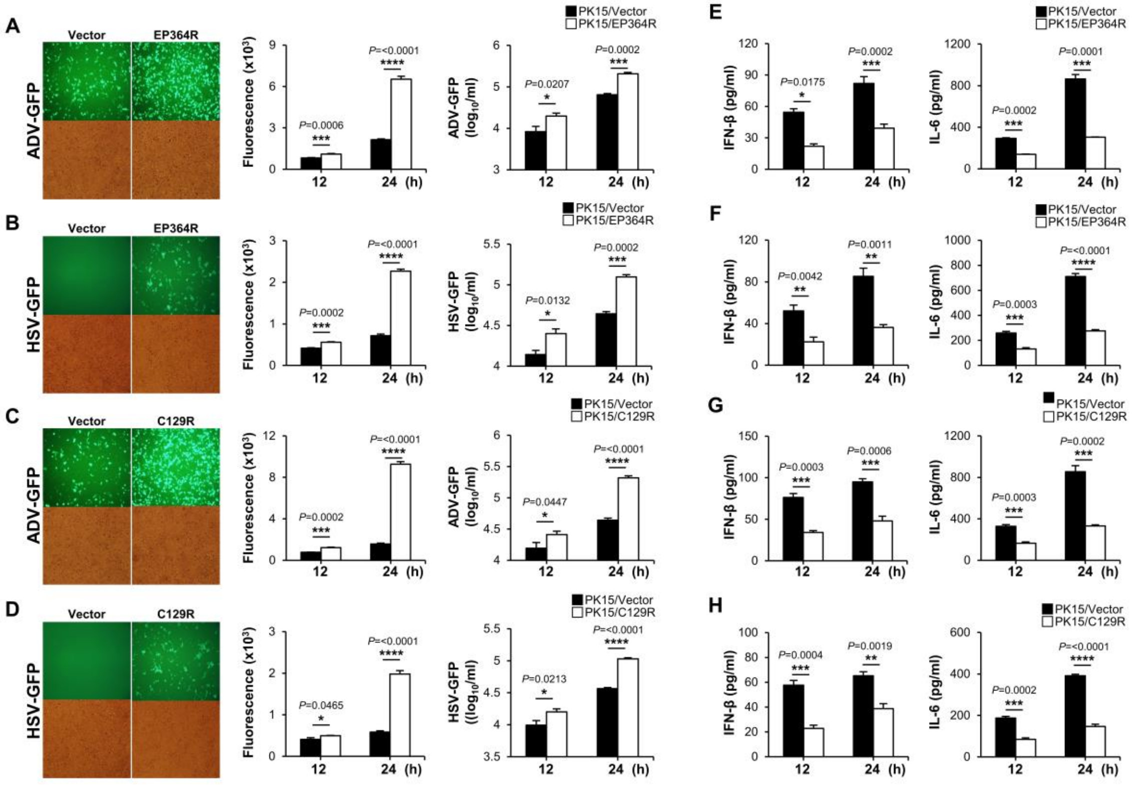

FIG S3 EP364R and C129R negatively regulate antiviral immune responses in PK-15 cells

PK-15 cells were transiently transfected with Flag-EP364R (A and B) or Flag-C129R (C and D) plasmids along with the control vector followed by the ADV-GFP (1MOI) (A and C) and HSV-GFP (1MOI) (B and D) infection at 24 hpt. Viral replication was visualized at 24 hpi by GFP expression levels using fluorescence microscopy and quantified at 12 hpi and 24 hpi by a fluorescence modulator. Virus titers of each sample were determined by plaque assay in A549 cells. Porcine IFN-β and IL-6 secretion in cell culture supernatant at 12 hpi and 24 hpi were determined by ELISA (E-H). Data are representative of at least two independent experiments, each with similar results, and the values are expressed as mean ± SD of three biological replicates. Student's t-test; * $p < 0.05$; ** $p < 0.01$; *** $p < 0.001$.

FIG S4

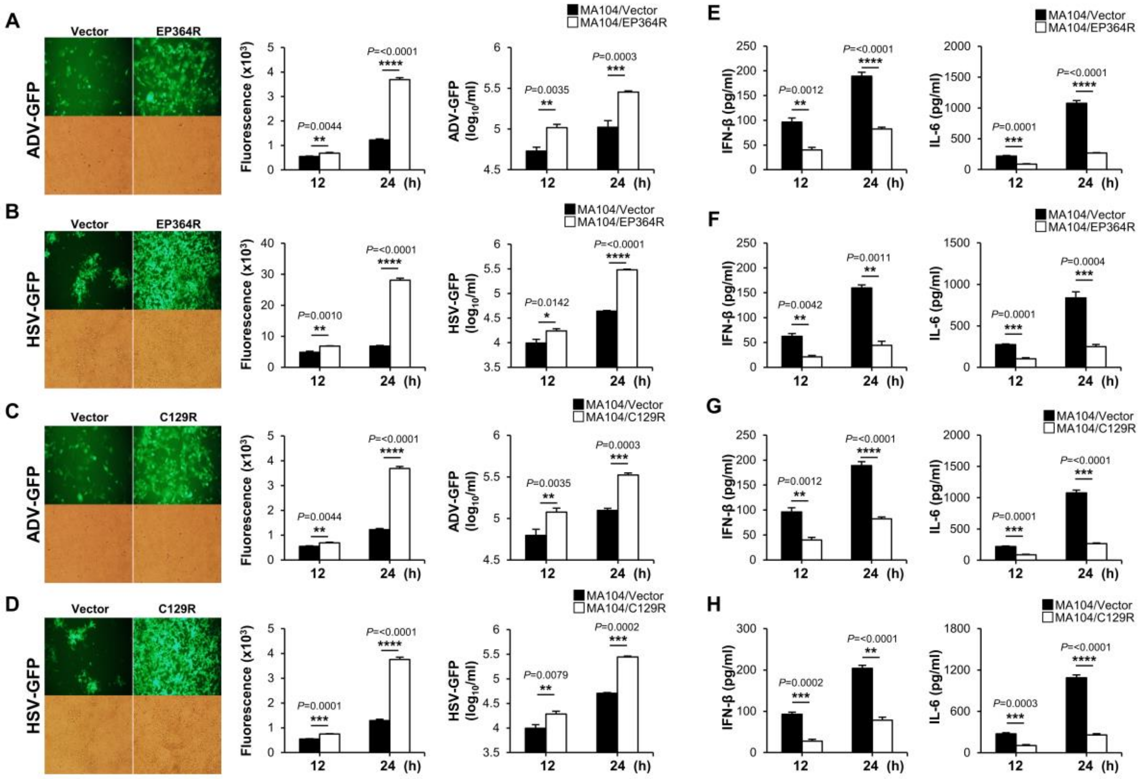

FIG S4 EP364R and C129R negatively regulate antiviral immune responses in MA104 cells

MA104 cells were transiently transfected with Flag-EP364R (A and B) or Flag-C129R (C and D) plasmids along with the control vector followed by the ADV-GFP (1MOI) (A and C) and HSV-GFP (1MOI) (B and D) infection at 24 hpt. Viral replication was determined at 24 hpi by GFP expression levels using fluorescence microscopy and quantified at 12 hpi and 24 hpi by fluorescence modulator. Virus titers of each sample were determined by plaque assay in Vero cells. Human IFN-β and IL-6 secretion in cell culture supernatant at 12 hpi and 24 hpi were determined by ELISA (E-H). Data are representative of at least two independent experiments, each with similar results, and the values are expressed as mean ± SD of three biological replicates. Student's t-test; * $p < 0.05$; ** $p < 0.01$; *** $p < 0.001$.

FIG S5

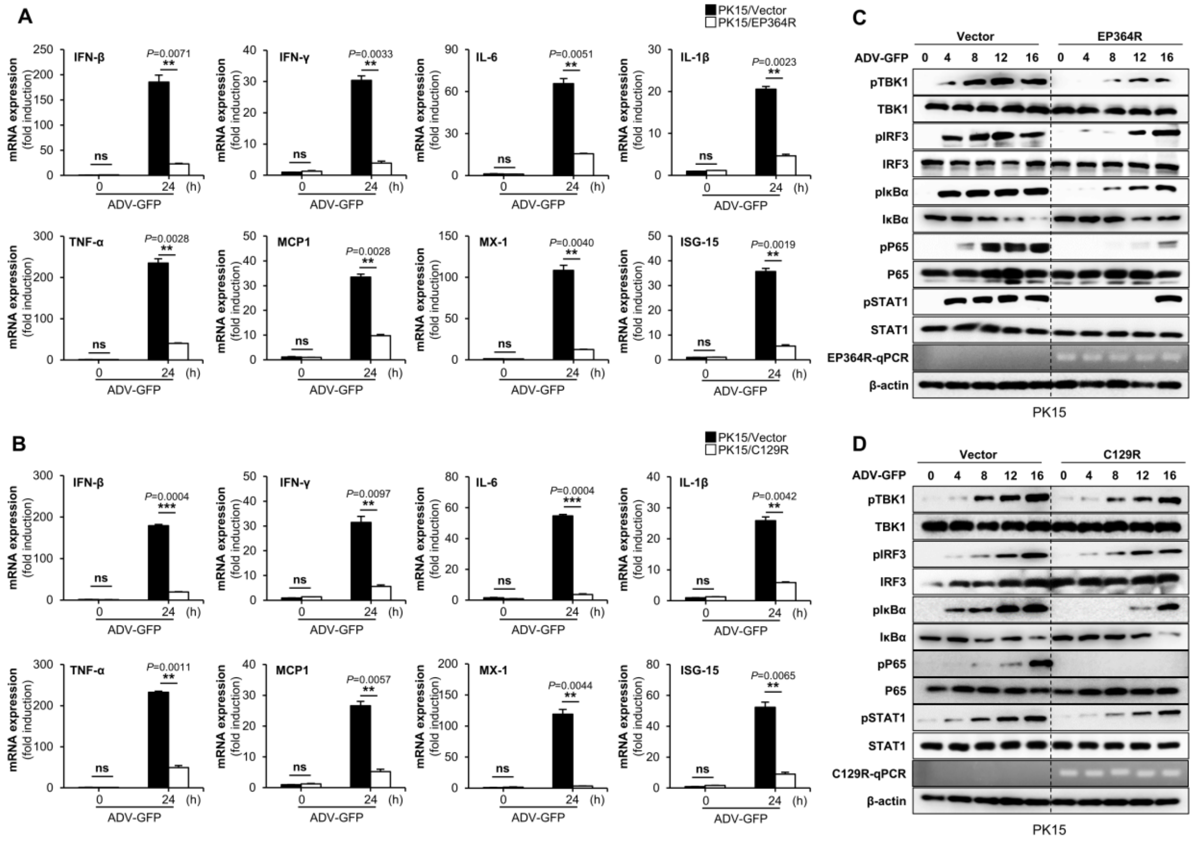

FIG S5 EP364R and C129R suppress cGAS-STING pathway and antiviral gene transcription in PK-15 cells

(A and B) PK-15 cells were transfected with Flag-EP364R or Flag-C129R plasmids and control vector plasmid. Cells were infected with ADV-GFP (1MOI) at 24 hpt, and cells were harvested at indicated time points. Then total RNA was extracted and analyzed the transcription of the indicated genes mRNA by quantitative real-time PCR. Data are representative of at least two independent experiments. (C and D) Flag-EP364R or Flag-C129R and control plasmids were transfected into PK-15 cells. Next, cells were infected with ADV-GFP (2MOI) and harvested at indicated time points. EP364R or C129R gene expression level, total and phosphorylated TBK1, IRF3, IκBα, p65, and STAT1 were measured by immunoblotting. β-actin was used as a loading control indicator.

All the data are representative of at least two independent experiments, each with similar results, and the values are expressed as mean ± SD of two biological replicates. All the immunoblot data are representative of at least two independent experiments, each with similar results. Student's t-test; * $p < 0.05$; ** $p < 0.01$; *** $p < 0.001$; ns, not significant.

FIG S6

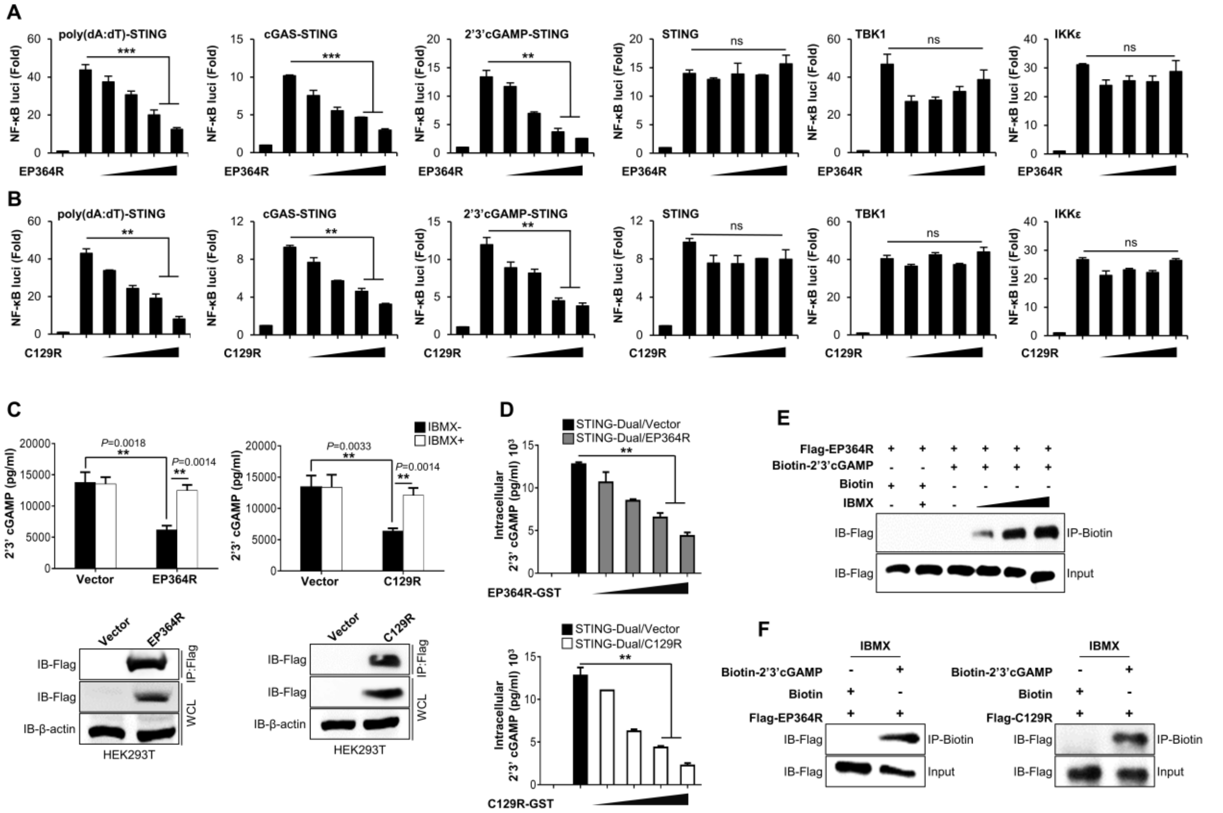

FIG S6 EP364R and C129R bind to 2'3'cGAMP and induce its degradation

(A and B) HEK293T cells were co-transfected with an increasing dose of Flag-EP364R or Flag-C129R together with firefly luciferase reporter plasmid encoding the NF-κB promoter plus TK renilla plasmid and cGAS, STING, TBK1, and IKKε or stimulated with poly(dA:dT) or 2'3'cGAMP for 24 h and 12 h respectively. TK-Renilla was used as transfection control to normalize firefly luciferase activity. STING overexpressed 293-Dual™ hSTING-A162 cells were used for poly(dA:dT), cGAS, and cGAMP induced luciferase assays. Results are expressed relative to those of Renilla luciferase alone (internal control). (C) *In vitro* 2'3'cGAMP degradation assay. Flag-EP364R or Flag-C129R proteins plus 2.5 μM 2'3'cGAMP with 1 mM IBMX were incubated for 22 h at 37^{0}C in reaction buffer followed by 2'3'cGAMP ELISA.. (D) 293-Dual™ hSTING-

A162 cells were co-transfected with indicated plasmids with cGAS plasmid Cells were harvested at 24 hpt and intracellular 2'3'cGAMP level were measured. . (E) IBMX inhibitor dose selection assay. Purified Flag-EP364R protein 2 μg was incubated with 10 μM biotin 2'3'cGAMP or 10 μM biotin with reaction buffer for 2 h at 30^{0}C with 0.25 mM, 0.5 mM, 1 mM, and 2 mM IBMX and subjected to biotin pulldown by streptavidin magnetic beads followed by immunoblotting with anti-Flag antibody. (F) ASFV Flag-tagged-EP364R or C129R protein interaction with biotin-conjugated 2'3'cGAMP. 2 μg of ASFV proteins were incubated with 10 μM biotin 2'3'cGAMP or 10 μM biotin with reaction buffer for 2 h at 30^{0}C with 1 mM IBMX and subjected to biotin pulldown by streptavidin magnetic beads followed by immunoblotting with anti-Flag antibody. All the luciferase data are representative of at least two independent experiments, each with similar results, and the values are expressed as mean ± SD of two biological replicates. All the ELISA data are representative of at least two independent experiments, each with similar results, and the values are expressed as mean ± SD of three biological replicates. All the immunoblot data are representative of at least two independent experiments, each with similar results. Student's t-test; * $p < 0.05$; ** $p < 0.01$; *** $p < 0.001$.

FIG S7

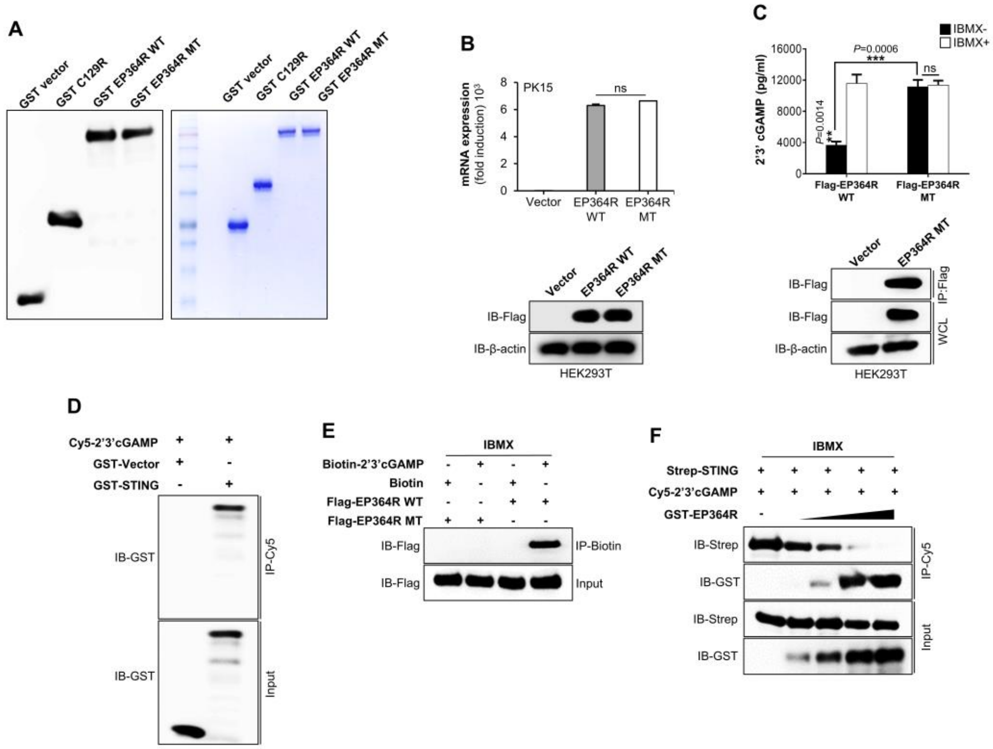

FIG S7 GST protein purification and STING-cGAMP interaction competition

(A) Purified GST protein was confirmed by western blot analysis and comassie staining of SDS-PAGE. (B) Overexpression of Flag-EP364R-WT and Flag-EP364R-MT in PK-15 cells and HEK293T cells. PK-15 cells were stably transfected with an Flag vector or Flag-EP364R-WT and Flag-EP364R-MT and cDNA synthesized for qRT-PCR. Overexpression of Flag-EP364R-WT and its mutant plasmid was confirmed by HEK293T cell stably transfected with an IRES-Flag vector or Flag-EP364R-WT Flag-EP364R-MT immunoblotting with anti-Flag antibodies and β-Actin was used to confirm equal loading of proteins. (C) *In vitro* 2'3'cGAMP degradation assay. Flag-EP364R-WT or its mutant EP364R-MT proteins plus 2.5 μM 2'3'cGAMP with 1 mM IBMX were incubated 22 h at 37^{0}C in reaction buffer followed by 2'3'cGAMP ELISA to quantify the remaining

2'3'cGAMP level in incubated samples. (D) *In vitro* STING – 2'3'cGAMP binding assay. Immunoprecipitated 2 μg of Strep-STING protein were incubated with 10 μM of Cy5 conjugated 2'3'cGAMP with reaction buffer for 2 h at 30^0C with 1 mM IBMX and subjected toCy5 pulldown followed by immunoblotting with antiGST antibody. (E) *In vitro* 2'3'cGAMP binding assay. Flag immunoprecipitated 2 μg of ASFV EP364R-WT or EP364R-MT proteins were incubated in 10 μM 2'3'cGAMP-biotin or 10 μM biotin with reaction buffer for 2 h at 30^0C with 1 mM IBMX followed by streptavidin pulldown (F) *In vitro* 2’3’cGAMP binding competition assay. The increasing amount of GST purified protein of EP364R-WT (0.25 μg, 0.5 μg, 1 μg, 2 μg) and GST vector protein as control (1.75 μg, 1.5 μg, and 1 μg) with a constant amount of Strep-STING protein (1 μg) incubated with 10 μM Cy5 conjugated 2'3'cGAMP in reaction buffer for 2 h at 30^0C with1mM IBMX. Incubated protein mixture immunoprecipitated by anti Cy5 antibody followed by immunoblotting with anti-GST and anti-Strep antibodies. All the ELISA data are representative of at least two independent experiments, each with similar results, and the values are expressed as mean ± SD of three biological replicates. All the immunoblot data are representative of at least two independent experiments, each with similar results. Student’s t-test; * $p < 0.05$; ** $p < 0.01$; *** $p < 0.001$; ns, not significant.

164
